# Time-dependent effects of BRAF-V600E on cell cycling, metabolism, and function in engineered myocardium

**DOI:** 10.1126/sciadv.adh2598

**Published:** 2024-01-24

**Authors:** Nicholas Strash, Sophia DeLuca, Geovanni L. Janer Carattini, Yifan Chen, Tianyu Wu, Abbigail Helfer, Jacob Scherba, Isabella Wang, Mehul Jain, Ramona Naseri, Nenad Bursac

**Affiliations:** ^1^ Department of Cell Biology, Duke University, Durham NC, USA.; ^2^ Department of Biomedical Engineering, Duke University, Durham NC, USA.

## Abstract

Candidate cardiomyocyte (CM) mitogens such as those affecting the extracellular signal–regulated kinase (ERK) signaling pathway represent potential targets for functional heart regeneration. We explored whether activating ERK via a constitutively active mutant of B-raf proto-oncogene (BRAF), *BRAF-V600E* (caBRAF), can induce proproliferative effects in neonatal rat engineered cardiac tissues (ECTs). Sustained CM-specific caBRAF expression induced chronic ERK activation, substantial tissue growth, deficit in sarcomeres and contractile function, and tissue stiffening, all of which persisted for at least 4 weeks of culture. caBRAF-expressing CMs in ECTs exhibited broad transcriptomic changes, shift to glycolytic metabolism, loss of connexin-43, and a promigratory phenotype. Transient, doxycycline-controlled caBRAF expression revealed that the induction of CM cycling is rapid and precedes functional decline, and the effects are reversible only with short-lived ERK activation. Together, direct activation of the BRAF kinase is sufficient to modulate CM cycling and functional phenotype, offering mechanistic insights into roles of ERK signaling in the context of cardiac development and regeneration.

## Teaser

Expression of the kinase BRAF in cardiomyocytes promotes cell cycle activation.

## INTRODUCTION

Myocardial infarction results in the permanent loss of candidate cardiomyocytes (CMs) and decline in heart function. Promising strategies to replace the lost heart tissue and improve cardiac function include cell therapies to engraft functional CMs differentiated from pluripotent stem cells (PSCs) (*1*–*3*) or gene therapies to drive endogenous CM proliferation (*4*). Understanding the signaling pathways that can stimulate CM proliferation is of paramount importance for both therapeutic strategies as cardiac structural and functional recovery is expected to be directly proportional to the numbers of exogenously engrafted or endogenously proliferated CMs.

The mitogen-activated protein kinase (MAPK) signaling pathway is a widely studied, complex signaling pathway that governs multiple biological processes essential for both embryonic development and maintenance of tissue homeostasis (*5*–*8*). A number of human cancers have at least one mutation in a component of the MAPK signaling cascade resulting in dysregulation of its primary canonical effectors, extracellular signal–regulated kinases (ERKs) (*9*, *10*). In healthy cells, ERK activity is initiated by upstream kinases, which are influenced by a variety of extracellular and intracellular signaling molecules. Constitutive activation of ERK in response to transient or sustained growth factor stimulation is prevented by inhibitory negative feedback that tightly regulates ERK activity (*11*, *12*). BRAF, a frequently mutated protein in multiple cancer types, is a serine/threonine protein kinase within the canonical MAPK pathway that is responsible for regulating the MAPK kinase (MEK), which, in turn, activates ERK. The most common activating somatic mutation of BRAF is the V600E mutation, which is known to evade negative feedback inhibition and, as a result, is highly tumorigenic (*13*). Since BRAF is thought to only activate the MEK/ERK signaling axis (*14*), *BRAF-V600E* (caBRAF) can be used as a means to study sustained activation of ERK due to evasion of negative regulatory feedback mechanisms (*11*).

When activated in the heart postnatally or in vitro, ERK has been paradoxically characterized as a promoter of both CM hypertrophy (*7*, *15*–*19*) and proliferation (*20*–*23*). ERK dysregulation in the heart during human development has been linked to congenital cardiac defects characterized by concentric organ hypertrophy, as shown in patients with Noonan syndrome, Costello syndrome, and cardiofaciocutaneous syndrome (*6*). However, at the cellular level, this hypertrophic phenotype seems to be variable and mutation dependent as CM hyperplasia rather than hypertrophy has been reported in multiple patients and mouse models with RASopathy (*24*–*27*). In vitro, patient-derived human induced PSC (hiPSC)–CMs with germline BRAF-activating mutations (T599R and Q257R) displayed a phenotype reflective of hypertrophic cardiomyopathy (*28*, *29*), while in vivo mouse experiments have shown that transgenic CM–specific *BRAF-V600E* expression is sufficient to drive cardiac hypertrophy (*16*) and that BRAF is an important mediator of CM response to prohypertrophic stimuli (*30*).

In vitro three-dimensional (3D) engineered cardiac tissue (ECT) models represent a versatile medium-throughput tool for studies of CM development, maturation (*31*–*35*), and disease (*36*–*38*), including identification of potential CM mitogens for use in cardiac regeneration (*39*–*41*). Compared to standard monolayer culture, CMs in 3D ECTs show improved maturation and reduced cycling (*42*). In addition, ECTs enable improved studies of cell-autonomous effects in the heart without multiple confounding factors present in vivo. In this report, we used a 3D neonatal rat ventricular myocyte (NRVM) ECT culture system exhibiting advanced maturation and function (*42*–*44*) to study the structural and functional effects of targeted ERK activation induced by CM-specific lentiviral expression of caBRAF. We observed proproliferative and antimaturation effects on CMs that yielded substantial tissue growth and functional deficit in ECTs lasting for at least 4 weeks. RNA sequencing analysis of control and caBRAF tissues revealed broad transcriptomic differences in cell metabolism and cell-matrix interactions that underlie the observed functional changes. Depending on its duration, doxycycline (dox)–inducible transient expression of caBRAF yielded reversible or long-lasting functional and proliferative effects. Our in vitro studies suggest that sustained ERK activity can counter the natural maturation of postnatal CMs, yielding a progrowth phenotype of potential relevance for congenital heart diseases and development of cardiac regenerative therapies.

## RESULTS

### caBRAF expression promotes cell cycling, morphological changes, and functional deficit in NRVM ECTs

**Fig. 1.**
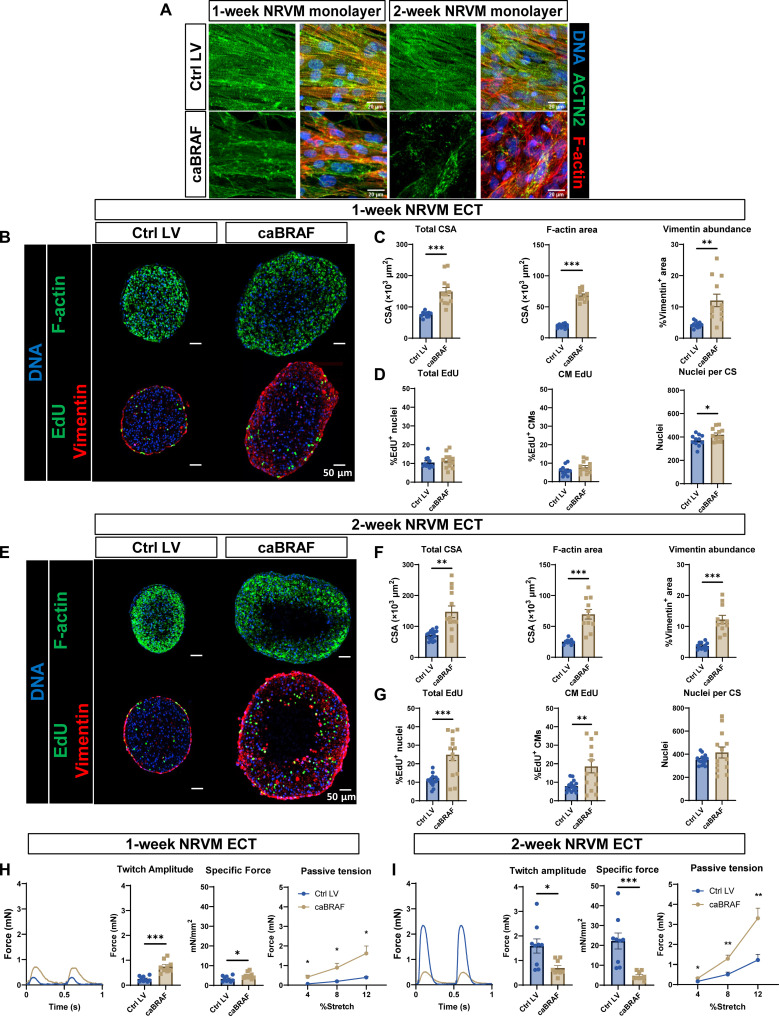
caBRAF expression induces morphological, cell cycling, and functional changes in NRVM ECTs with time of culture. (**A**) Representative images of (left) 1-week and (right) 2-week NRVM monolayers showing the deteriorating effects of caBRAF expression on sarcomere structure. (**B** to **G**) Representative images of NRVM ECT cross sections and corresponding morphological and cell cycling quantifications after 1 week (B to D) and 2 weeks (E to G) of culture. (**H** and **I**) Representative twitch force traces at 2-Hz stimulation, twitch amplitudes, specific forces (force per CSA), and passive tension–length relationships in ECTs after 1 week (H) and 2 weeks (I) of culture. %Stretch values are shown relative to the initial testing length. Data: *n* = 10 to 12 ECTs (C and D), *n* = 11 to 15 ECTs (F and G), and *n* = 9 ECTs (H and I). Column graphs showing individual data points, means ± SEM; line plots, means + SEM. *P < 0.05, **P < 0.01, and ***P < 0.001 versus Ctrl LV. All experiments were repeated in *N* = 3 independent ECT batches.

To ensure CM-specific transgene expression, we generated lentiviruses (LVs) in which the muscle-specific MHCK7 (chimeric α-MHC and murine creatine kinase) promoter (*45*) drove expression of mCherry control (Ctrl) or caBRAF-2A-mCherryNLS (fig. S1, A and B). We first examined the effect of LV-expressed caBRAF in NRVM monolayers and found that sarcomere organization in CMs was disrupted by 1 week of culture and further deteriorated by 2 weeks of culture (Fig. 1A). In a 3D NRVM ECT system, CM-specific expression of caBRAF was associated with larger-tissue cross-sectional area (CSA) by 1 week (Fig. 1, B and C), and this morphological change persisted at 2 weeks of culture, at which point formation of a central acellular core was apparent (Fig. 1, E and F). As previously shown (*43*, *46*), in Ctrl ECTs, aligned cardiomyocytes strongly expressing F-actin, but not vimentin, resided in the tissue interior and were surrounded by an outer layer of vimentin^+^ fibroblasts (Fig. 1, B and E). caBRAF transduction yielded the occurrence of vimentin^+^ staining throughout the interior of the tissue, suggesting that caBRAF induced ectopic expression of vimentin in cardiomyocytes, consistent with the observed correlation between ERK activation and vimentin transcription shown in breast carcinoma (*47*). To determine whether caBRAF also drives increased cell cycle activation in NRVM ECTs, we delivered a pulse of 10 μM 5-ethynyl-2′-deoxyuridine (EdU) for 48 hours before tissue fixation and observed no difference in S phase entry at 1 week (Fig. 1D) but increased total and CM-specific EdU incorporation at 2 weeks of culture (Fig. 1G and fig. S1C), accompanied by increased expression of a mitosis marker phosphorylated histone H3 (fig. S1C). Together, caBRAF expression led to rapid morphological changes in NRVMs associated with increased intermediate filament production and cell cycle activation.

To explore functional consequences of caBRAF expression, we measured contractile function of NRVM ECTs using a custom force measurement system (*42*, *43*, *48*). After 1 week of culture, caBRAF ECTs showed increased contractile force (twitch) and specific force (force per CSA) amplitudes compared to Ctrl tissues (Fig. 1H). However, by 2 weeks of culture, caBRAF ECTs displayed significant decreases in both absolute (~2.3-fold) and specific (~4.7-fold) force compared to Ctrl ECTs (Fig. 1I), which normally increase in contractile strength over the 2-week culture period, indicative of functional maturation (*42*, *43*). As in monolayer studies, sarcomere structure in caBRAF-expressing ECTs was disrupted at both 1 and 2 weeks (fig. S1D), which likely contributed to the development of the contractile deficit with time of culture. In addition, caBRAF ECTs exhibited significantly increased passive tension at both 1 and 2 weeks of culture (Fig. 1, H and I), similar to the ERK-dependent tissue stiffening observed in our previous study with caERBB2 (Erb-B2 receptor tyrosine kinase 2, HER2) overexpression (*41*). Last, the observed contraction deficit was accompanied by slower twitch kinetics evident from both increased twitch rise and decay times that already showed changes after 1 week of culture (fig. S1, E and F). We then assessed whether other aspects of CM function were dysregulated by caBRAF expression and found reduced abundance of the gap junctional protein connexin-43 in both 1- and 2-week caBRAF tissues (fig. S2A). This was accompanied by significant slowing of action potential conduction and no change in action potential duration (fig. S2B). Collectively, in addition to substantial morphological changes, caBRAF expression induced significant functional deficits in NRVM ECTs that developed by 2 weeks of culture and were evident from the lower magnitude and slower kinetics of force generation, increased tissue stiffness, and reduced velocity of action potential propagation.

### Phenotypic changes induced by caBRAF expression in NRVM ECTs persist and increase during prolonged culture

We then determined whether the caBRAF-induced phenotype at 1 and 2 weeks of culture persisted longer term. We found that compared to Ctrl ECTs, all morphological indices including F-actin^+^ CSA, total CSA, and vimentin expression remained increased in caBRAF versus Ctrl tissues at 3 and 4 weeks of culture (fig. S3, A, B, D, and E). Moreover, while the EdU incorporation appeared to decrease over time compared to the earlier time points, caBRAF ECTs retained significantly higher rates of cell cycling at both 3 and 4 weeks of culture (fig. S3, C and F), with nucleus numbers per cross section (CS) progressively increasing over time. There was increased but constant abundance of vimentin^+^ area in caBRAF ECTs along with the increasing number of nuclei per CS, indicating that fibroblast proliferation is also increased by caBRAF expression such that the proportion of fibroblasts remains consistent in the ECT over time. In addition, caBRAF tissues showed persistent sarcomere disassembly at 3 and 4 weeks, with increased smooth muscle actin (ACTA2) expression observed at later time points (fig. S4A). We also found that contractile function of ECTs remained negatively affected by caBRAF expression, including the persistent increase in the tissue stiffness (fig. S3, G and H). Twitch kinetics of caBRAF ECTs was further slowed with time of culture, with caBRAF ECTs showing increased duration from 288 ± 5 ms at 2 weeks to 374 ± 22 ms at 4 weeks, versus Ctrl ECTs that exhibited stable twitch duration (189 ± 3 and 175 ± 10 ms at 2 and 4 weeks, respectively; fig. S4, B and C). Therefore, with prolonged culture, structural and morphological differences between caBRAF and Ctrl NRVM ECTs were maintained or even increased (for nucleus number, twitch decay time, and passive tension) (fig. S5). These long-term culture experiments also showed that caBRAF expression induced not only CM cycling but also fibroblast proliferation, as we observed increased number of nuclei in the vimentin^+^ ECT exterior (fig. S5), which we showed is highly enriched in fibroblasts (*43*).

### Bulk RNA sequencing reveals broad transcriptomic changes in CMs caused by caBRAF expression

**Fig. 2.**
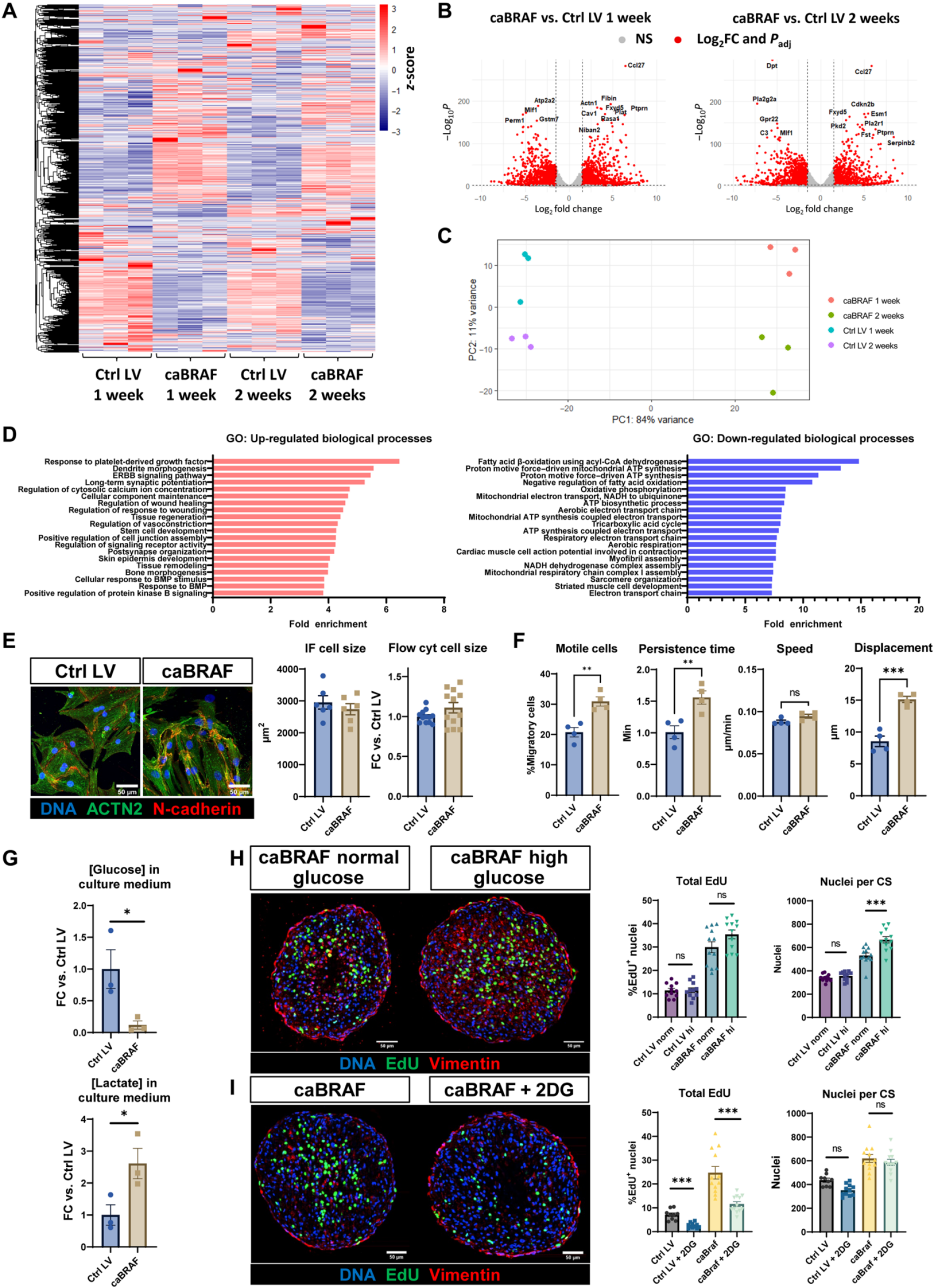
Transcriptomic analysis of NRVM ECTs reveals effects of caBRAF expression on CM migration and metabolism. (**A** to **D**) RNA sequencing analysis showing (A) *z*-scores and clustering of all differentially expressed genes across groups, (B) volcano plots showing cutoff points for classifying a gene as differentially expressed [absolute value of log_2_FC (log_2_ fold change) > 1.5 and *P*_adj_ < 0.01], (C) principal components (PC) analysis clustering analysis, and (D) GO analysis of the top up-regulated and down-regulated biological processes (at 2 weeks) in caBRAF versus Ctrl ECTs. BMP, bone morphogenetic protein; CoA, coenzyme A; ATP, adenosine 5′-triphosphate; NADH, reduced form of nicotinamide adenine dinucleotide (oxidized form). (**E**) Representative images of 1-week NRVM monolayers (left) used for quantification of CM size (right). IF, immunofluorescence. (**F**) Quantified parameters from cell migration analysis performed using live imaging of 1-week NRVM monolayers. (**G**) Glucose and lactate concentrations quantified in culture medium of 1-week NRVM ECTs. (**H** and **I**) Representative images of 2-week NRVM ECTs cultured in medium with (H) a normal or high glucose concentration and with (I) 2-deoxyglucose (2DG) treatment and corresponding quantifications of cell cycle activity. Data: *n* = 6 to 12 ECTs (E), *n* = 4 monolayers (F), *n* = 3 ECTs (G), *n* = 11 to 12 ECTs (H), and *n* = 9 to 12 ECTs (I). Column graphs showing individual data points, means ± SEM. *P < 0.05, **P < 0.01, and ***P < 0.001 versus indicated group. ns, not significant. All experiments were repeated in *N* = 3 to 4 independent ECT batches.

To gain further insights in molecular changes induced by caBRAF expression, we performed bulk RNA sequencing on caBRAF and Ctrl ECTs at 1 and 2 weeks of culture (Fig. 2). Broad differential clustering of expressed genes between Ctrl and caBRAF tissues was apparent in heatmaps at both culture times (Fig. 2A). Specifically, 2525 and 2214 genes were differentially expressed between caBRAF and Ctrl ECTs in 1- and 2-week samples, respectively (Fig. 2B). Performing gene ontology (GO) analysis on these differentially expressed genes revealed that, as expected, the GO terms MAPK signaling pathway and cell population proliferation were up-regulated, as well as epithelial-mesenchymal transition and extracellular matrix (ECM)-receptor interactions, including multiple matrix metalloproteinases and integrins (fig. S6A). On the other hand, consistent with the observed functional deficit (Fig. 1), genes involved in cardiac muscle development, oxidative phosphorylation, and heart contraction were down-regulated. We also found dysregulation of major ion channel, adenosine triphosphatase, and regulatory genes involved in CM electrical activity and calcium handling, including decreased expression of the sarcoplasmic/endoplasmic reticulum ATPase (*Atp2a2*), ryanodine receptor (*Ryr2*), and phospholamban (*Pln*) (fig. S6B). *Gja1* levels were not altered with caBRAF expression (fig. S6B), suggesting that the observed loss of connexin-43 protein (fig. S2A) was induced by a posttranscriptional regulatory mechanism.

By performing principal components analysis, we further found that a majority of the variance between samples occurred because of caBRAF expression rather than culture duration (Fig. 2C). Consistently, GO analysis returned the same or very similar differentially expressed pathways at the 1- and 2-week time points; hence, we focused on the pathways revealed by analysis of the 2-week tissues. Within differential GO terms of highest significance, we identified many up-regulated processes related to development and locomotion (Fig. 2D), while down-regulated GO terms pointed to profound metabolic changes, including decreased aerobic metabolism and mitochondrial function in caBRAF compared to Ctrl ECTs (Fig. 2D). We then performed ChEA3 (ChIP-X enrichment analysis 3) analysis (*49*), which revealed likely transcription factors that mediated observed transcriptomic differences between caBRAF and Ctrl ECTs (fig. S6C). Notably, transcription factors known to mediate cardiac mesodermal specification and early development such as *Meox1* (mesenchyme homeobox 1), *Tcf15* (transcription factor 15), *Gata4* (GATA binding protein 4), *Nkx2-5* (NK2 homeobox 5), and *Tbx20* (T-Box transcription factor 20) were implicated as likely drivers of the down-regulated genes (fig. S6C) (*50*). Together and consistent with our structural and functional studies, the transcriptomic analysis suggested that caBRAF expression predominantly induced antimaturation effects in NRVM ECTs, characterized by a switch to an early cardiac developmental program, increased DNA synthesis, ECM remodeling, and decreased contractile function.

An unexpected result of our transcriptomic analysis was the lack of an obvious cardiac hypertrophic signature, as ERK has been widely reported to mediate CM hypertrophy (*6*, *7*, *17*, *19*, *51*). To further explore whether caBRAF expression induced CM hypertrophy, we measured cell size in sparse NRVM monolayer cultures by quantitative immunostaining and found no size differences between caBRAF and Ctrl CMs, which was further confirmed using flow cytometry on NRVMs cultured in confluent monolayers (Fig. 2E). To additionally validate transcriptomic results, we performed live time-lapse microscopy imaging to quantify cell migration in NRVM monolayers since locomotion was a significantly up-regulated process in GO analysis (Fig. 2D). Quantification of nuclear motion in monolayers over 7 hours (fig. S7A) revealed an increased proportion of motile cells, cell persistence time, and displacement induced by caBRAF expression (Fig. 2F), which was further evident from consistently right-shifted histograms of cell migration parameters (fig. S7, B and C).

Since GO analysis suggested that aerobic metabolism was the most down-regulated process in caBRAF versus Ctrl ECTs, we additionally examined metabolic consumption rates of ECTs by measuring the glucose and lactate concentrations in spent culture medium. In caBRAF ECT medium, glucose concentration was decreased, and lactate concentration was increased, suggesting a shift favoring glycolytic metabolism (Fig. 2G), similar to findings in mice with CM-specific caERBB2 expression (*52*). To further examine whether caBRAF induced a reliance on glucose availability, we supplemented additional glucose in the culture medium and found that while Ctrl LV ECTs were not affected by the additional glucose, this environment better supported the glycolytic demand of caBRAF ECTs, evidenced by increased number of nuclei (Fig. 2H). To further support this observation, we treated ECTs with 2-deoxyglucose, a commonly used glycolysis blocker, and found that caBRAF-induced cell cycle entry is reliant on glycolytic activity (Fig. 2I). Together, caBRAF-increased CM migration, a metabolic shift toward glycolysis, and lack of CM hypertrophy, all suggested from the RNA sequencing analysis, were successfully confirmed in the described follow-up studies.

### Paracrine signals from caBRAF-expressing CMs contribute slower contraction kinetics of NRVM ECTs but not other observed phenotypic changes

**Fig. 3.**
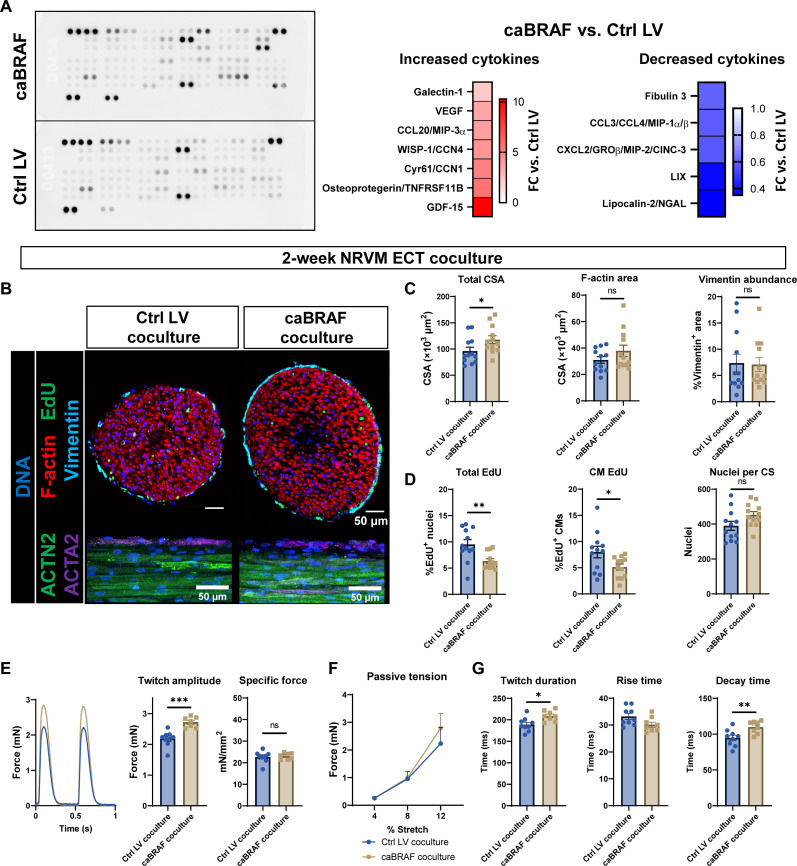
Secreted factors from caBRAF ECTs are minor contributor to caBRAF expression–induced phenotype. (**A**) Image of a qualitative cytokine array used to analyze culture medium from 1-week NRVM ECTs and corresponding quantifications of relative cytokine expressions in caBRAF versus Ctrl LV group. VEGF, vascular endothelial growth factor; MIP-3α, macrophage inflammatory protein 3α; CCL20, C-C motif chemokine ligand 20; WISP-1, cellular communication network factor 4; Cyr61, cellular communication network factor 1; GROβ, C-X-C motif chemokine ligand 2; CINC-3, cytokine-induced neutrophil chemoattractant 3; LIX, C-X-C motif chemokine ligand 5; NGAL, lipocalin 2. (**B** to **D**) Representative cross section and whole-mount images (B) and corresponding quantifications of morphological (C) and cell cycling (D) parameters in nontransduced NRVM ECTs cocultured for 2 weeks with either Ctrl LV or caBRAF ECTs. (**E** to **G**) Representative twitch force traces at 2-Hz stimulation and corresponding quantifications of twitch amplitudes and specific forces (E), passive tension–length relationships (F), and twitch kinetic parameters (G) in nontransduced NRVM ECTs cocultured for 2 weeks with either Ctrl LV or caBRAF ECTs. %Stretch values are shown relative to the initial testing length. Data: *n* = 12 ECTs (C and D) and *n* = 7 to 9 ECTs (E to G). Column graphs showing individual data points, means ± SEM. Line plots, means + SEM. *P < 0.05, **P < 0.01, and ***P < 0.001 versus Ctrl LV. All experiments were repeated in *N* = 3 independent ECT batches.

A previous study suggested that paracrine factors from nonmyocytes expressing mutated BRAF contribute to hiPSC-CM hypertrophy in a transforming growth factor–β–dependent manner (*28*). This prompted us to examine the potential roles of paracrine signaling in driving the phenotypic changes in NRVMs observed with caBRAF expression. We first assessed differentially secreted cytokines in caBRAF ECTs at 1 week of culture using a qualitative cytokine array and found increases in growth and differentiation factor–15 (GDF-15) and osteoprotegerin (OPG), associated with transforming growth factor–β and tumor necrosis factor–α (TNFα) signaling, respectively, as well as WNT-associated cytokines CCN1 and CCN4 (cellular communication network factor 1 and 4) (Fig. 3A). To further assess potential paracrine and autocrine effects from caBRAF expression, we cultured a caBRAF or a Ctrl LV ECT with a nontransduced ECT in the same well and performed structural and functional assessments of the nontransduced ECT after 2 weeks of culture (*53*). Compared to coculture with Ctrl ECTs, coculture with caBRAF ECTs increased the CSA of nontransduced ECTs without inducing other morphological changes (Fig. 3, B and C ) and decreased the percentage of EdU^+^ CMs without changing total nucleus number (Fig. 3D). Functionally, compared to soluble factors from Ctrl ECTs, soluble factors from caBRAF ECTs led to an increase in contractile force generation in nontransduced ECTs but no increase in specific force amplitude (Fig. 3E) or tissue stiffness (Fig. 3F). Furthermore, coculture with caBRAF ECTs slightly increased twitch duration and decay time in nontransduced ECTs (Fig. 3G). To determine whether increased secretion of GDF-15 or OPG specifically was responsible for the observed paracrine effects on ECT function, we treated ECTs with these recombinant proteins for either the final 48 hours of culture (fig. S8A) or nearly the entire culture period (fig. S8B) but did not note any functional effects. Together, these studies suggested that soluble paracrine signals within caBRAF-expressing ECTs minimally contributed to the observed phenotype, which instead was directly caused by CM-autonomous, juxtacrine, or other microenvironmental effects.

### caBRAF induces increased ERK activity that is required for the onset of functional decline and maintenance of increased cell cycling

**Fig. 4.**
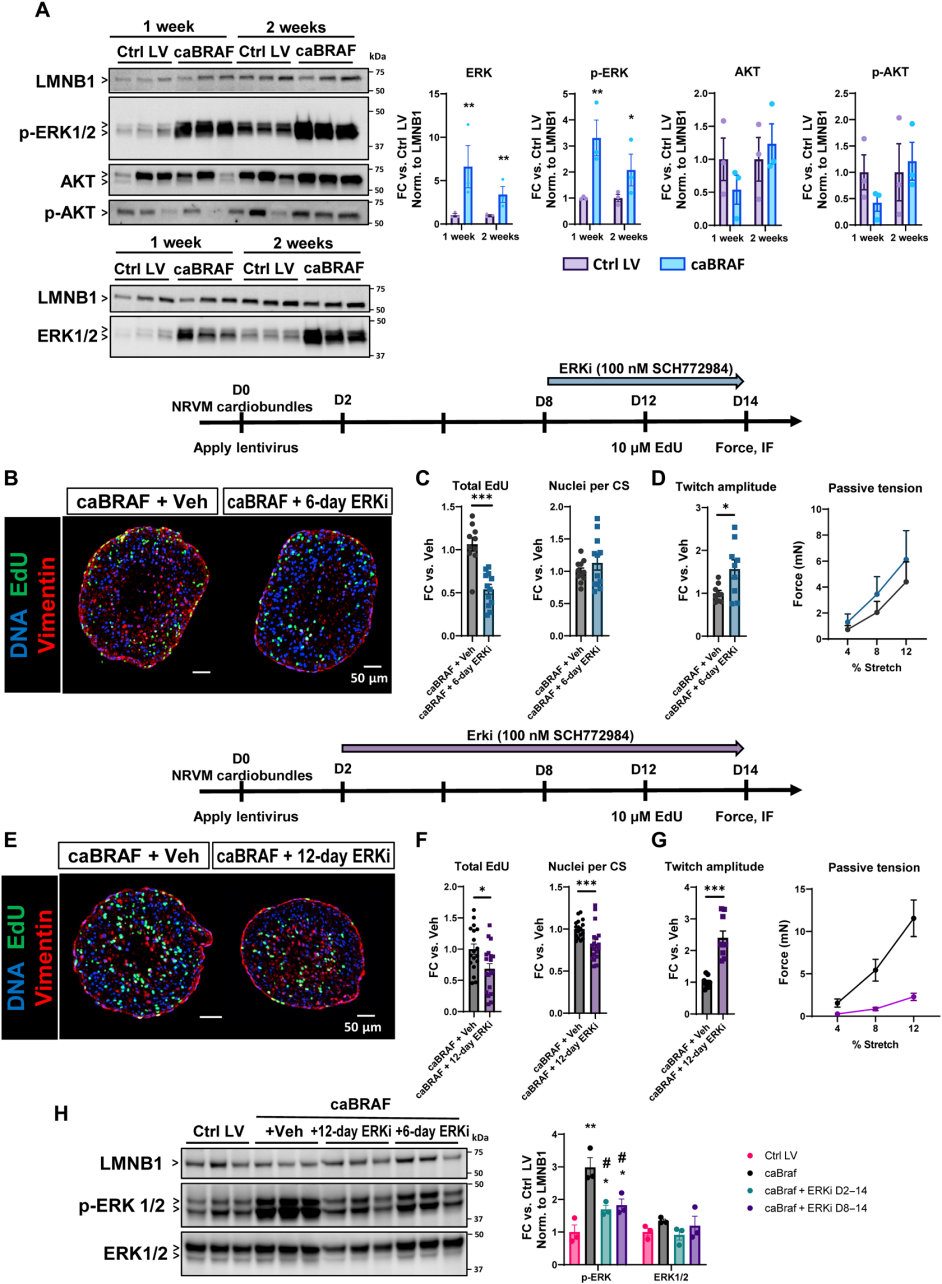
Elevated ERK activity in caBRAF ECTs is required for functional decline and increased cell cycle activity. (**A**) Western blots and corresponding quantifications of ERK and AKT activity in 2-week ECTs shown normalized to Lamin B1 (LMNB1) with arrowheads indicating the quantified protein bands. (**B** to **G**) Representative cross-sectional images (B and E) and corresponding quantifications of cell cycling and morphological parameters (C and F) and twitch amplitude and passive tension–length relationships (D and G) in 2-week ECTs treated on culture days 8 to 14 (B to D) or days 2 to 14 (E to G) with a vehicle solution or 100 nM SCH772984 (ERKi). (**H**) Representative Western blots and corresponding quantifications of ERK activity in 2-week ECTs from (B) to (G) shown normalized to LMNB1 with arrowheads indicating the quantified protein bands. Data: *n* = 3, each point is *n* = 3 ECTs pooled together (A and H), *n* = 9 ECTs (C and F), *n* = 10 to 12 ECTs (D), and *n* = 18 to 19 ECTs (G). Column graphs showing individual data points, means ± SEM. Line plots, means + SEM. *P < 0.05, **P < 0.01, and ***P < 0.001 versus Ctrl LV or caBRAF + vehicle (Veh). ^#^*P* < 0.05 vs caBRAF. All experiments were repeated in *N* = 3 to 5 independent ECT batches.

To confirm that ERK signaling was activated by caBRAF expression, we performed Western blot analysis and found significant increases in total and phosphorylated (p-) ERK in both the 1- and 2-week NRVM ECTs transduced with caBRAF, while AKT and p-AKT expression were not changed (Fig. 4A). We then explored whether the caBRAF-induced phenotype in NRVM ECTs could be reversed by ERK inhibition. Similar to our previous studies with caERBB2 expression (*41*), we applied 100 nM of the small molecule ERK inhibitor SCH772984 (ERKi) during the second week of ECT culture during which the functional deficit in caBRAF ECTs developed (Fig. 1). Compared to vehicle control, ERKi reduced cell cycling (Fig. 4C) without affecting other morphological parameters (fig. S9A), while also partially restoring contractile function (Fig. 4D) without rescuing increased passive tension, slowed twitch kinetics (fig. S9B), or sarcomere disassembly (fig. S9C). Overall, ERKi treatment during the second week of culture was only able to reverse some of the effects of caBRAF expression. This suggests that sustained elevated ERK activity is required to maintain the rate of cell cycle activation and declined tissue function but is dispensable for the other morphological parameters and tissue stiffness once established by 1 week in vitro (Fig. 1, B to D). We then asked whether increased ERK activity is required for the onset of the caBRAF phenotype by starting a 12-day ERKi treatment at culture day 2 and found reduced cycling (Fig. 4, E and F) and blunted morphological changes (fig. S9D) as well as rescued contractile function and passive tension (Fig. 4G). Furthermore, 12-day ERKi treatment prevented sarcomere disassembly (fig. S9F) but had no effect on the twitch kinetics (fig. S9E), which therefore appear to occur via an ERK-independent mechanism. We also found that p-ERK abundance remained elevated in both ERKi treatment groups relative to Ctrl LV, although this level was reduced compared to vehicle-treated caBRAF ECTs (Fig. 4H). Overall, increased ERK activity is required for the onset and maintenance of the caBRAF phenotype, and ERK inhibition alone is not sufficient to rescue the negative effects of caBRAF.

### Dox-inducible caBRAF expression reveals rapid onset kinetics and conditions for reversibility of caBRAF-induced phenotype

**Fig. 5.**
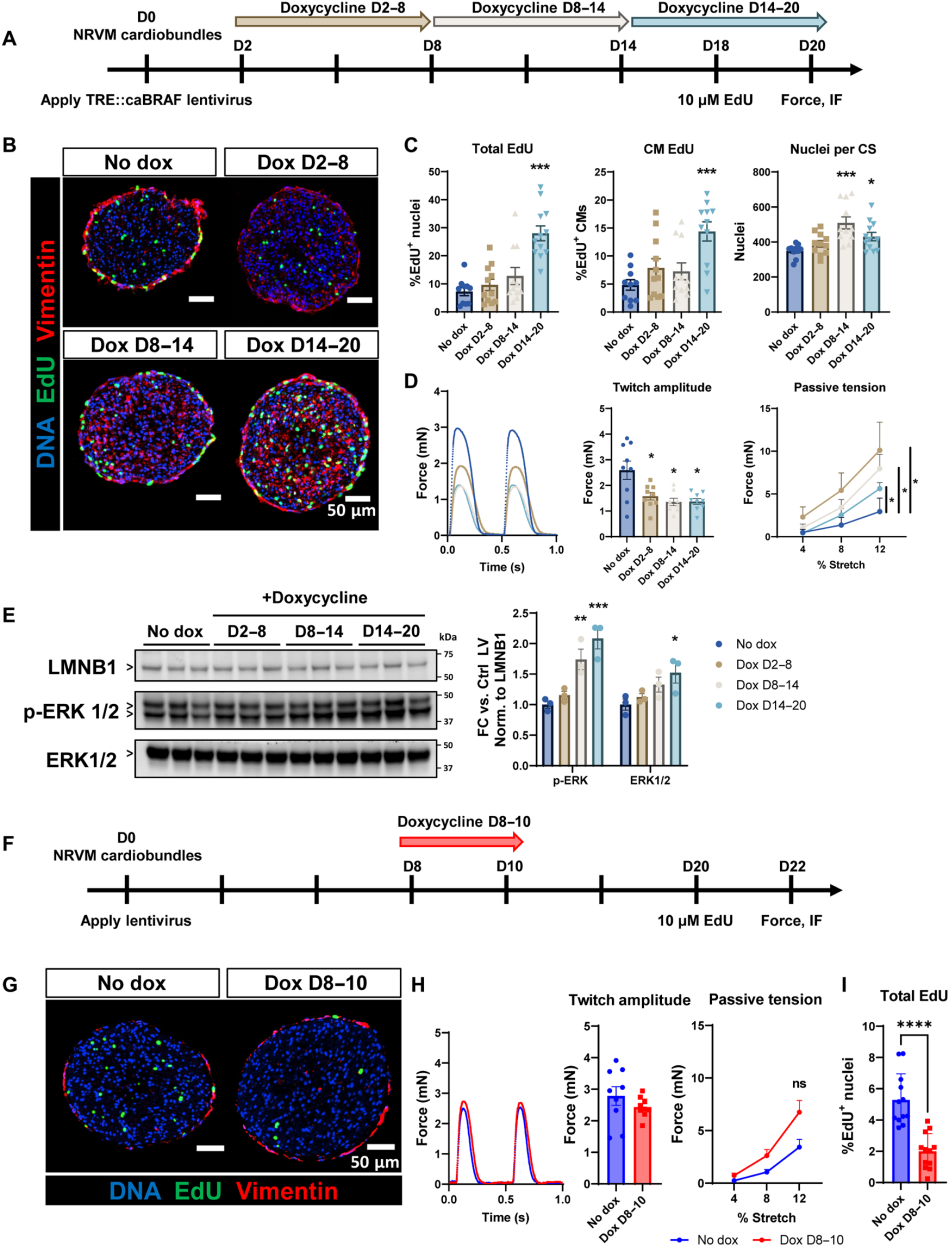
Dox-inducible expression of caBRAF reveals onset kinetics and duration of phenotypic changes in NRVM ECTs. (**A**) Schematic of experimental design for (B) to (E). (**B** to **D**) Representative cross-sectional images (B) and quantifications of cell cycle activity (C), twitch amplitude, and passive tension–length relationships (D) of 3-week NRVM ECTs treated with dox on the indicated days to induce caBRAF expression. (**E**) Western blots and corresponding quantifications of ERK activity in ECTs from (A) to (D) normalized to LMNB1 with arrowheads indicating the quantified protein bands. (**F**) Schematic of experimental design for (G) to (I). (**G** to **I**) Representative cross-sectional images of ECTs (G) and quantifications of twitch amplitude with representative force trace and passive tension–length relationships (H) and cell cycling (I). Data: *n* = 11 to 12 ECTs (C), *n* = 8 to 9 ECTs (D), *n* = 3, each point is *n* = 3 ECTs pooled together (E), *n* = 9 ECTs (H), and *n* = 12 ECTs (I). Column graphs showing individual data points, means ± SEM. Line plots, means + SEM. *P < 0.05, **P < 0.01, and ***P < 0.001 versus no dox. All experiments were repeated in *N* = 3 independent ECT batches.

To assess whether transiently expressed caBRAF can increase cardiomyocyte cell cycle activation without permanent functional decline, we constructed an all-in-one LV conferring dox-inducible, cardiomyocyte-specific caBRAF expression (fig. S10A). Transducing ECTs with this LV and then applying dox during second week of culture produced a similar phenotype to that resulting from constitutive caBRAF expression (Fig. 1), including increase in ECT size, EdU incorporation, contractile deficit, and tissue stiffness. On the other hand, Ctrl LV– and Ctrl LV + dox–treated control groups exhibited no structural or functional changes (fig. S10, B to E). To examine the duration and persistence of the caBRAF-induced phenotype following dox removal, we treated ECTs in either their first, second, or third week of a 3-week culture (Fig. 5A). We found that while DNA synthesis rates were only increased in tissues most recently treated with dox (Fig. 5C), contractile function was decreased, and passive tension increased for all dox-treated tissues (Fig. 5D). Similar to the EdU increase, p-ERK and ERK protein abundances were increased only in tissues most recently treated with dox (Fig. 5E). Consistent with persistently reduced contractile function, we observed sarcomere disorganization (fig. S11A) and aberrant twitch kinetics (fig. S11B) in all ECTs treated with dox. These results indicate that in the context of 6-day dox treatment, functional deficit in ECTs persists even in the absence of continued caBRAF expression, whereas proproliferative effects are transient and directly dependent on caBRAF expression.

We then investigated how rapidly caBRAF expression produced the observed phenotype by treating ECTs with dox from days 8 to 10, days 10 to 12, or days 12 to 14 with day 14 as the experimental end point (fig. S12A). We found that all dox-treated groups showed increased EdU incorporation (fig. S12, B and C), suggesting that increased cell cycling is an immediate phenotype that occurs with caBRAF expression. On the other hand, we observed decreased contractile force in ECTs treated from days 8 to 10 and days 10 to 12, but not from days 12 to 14 (fig. S12D), indicating that the contractile deficit appears later than the cell cycle entry, i.e., within ~4 days following activation of caBRAF expression. This functional deficit was associated with progressive loss of sarcomere structure evident from mild sarcomere disassembly (fig. S12F). We then assessed whether a longer, 12-day washout after 2-day dox treatment of ECTs would recover caBRAF-induced functional deficit and observed that these 2-day dox-treated tissues showed normal contractile function (Fig. 5H) and reduced EdU incorporation (Fig. 5, G and I). Overall, while a dox-induced 6-day caBRAF expression in cardiomyocytes appears to induce persistent functional deficits in ECTs, a shorter 2-day caBRAF expression results in transient cell cycle activation and reduction in contractile force (fig. S12), which can be recovered after caBRAF is turned off (Fig. 5H and fig. S13).

## DISCUSSION

The MAPK pathway is a complex signal transduction pathway with broad effects on cellular fate (*54*, *55*) and function (*20*, *56*). In this study, we sought to determine the molecular and functional effects of caBRAF-induced ERK activation in neonatal rat cardiomyocytes in the context of an in vitro engineered heart tissue model. The MAPK pathway and related ERK activity have been recently implicated in studies attempting to restore heart function after myocardial infarction via endogenous CM proliferation. Thus far, these studies involved activation of MAPK upstream signaling components such as cell receptors (caERBB2) (*23*, *57*) or their ligands (epidermal growth factor, fibroblast growth factor, and neuregulin 1) (*58*–*61*) but not the direct modulation of downstream components (BRAF and ERK) (*17*). Stimulation of upstream components of the pathway can activate not only the canonical RAF/RAS/MEK/ERK signaling axis but also parallel mitogenic pathways such as phosphatidylinositol 3-kinase/AKT/mammalian target of rapamycin (*62*, *63*). To examine more direct ERK-mediated effects on CMs, we expressed *BRAF-V600E* (caBRAF) in NRVMs to constitutively drive ERK activity. As a consequence, we observed broad transcriptomic changes associated with sustained CM cell cycle activity, a shift toward glycolytic metabolism, deteriorated sarcomeres, contractile deficit, tissue stiffening, cellular decoupling, and conduction slowing. In contrast, Ctrl NRVM tissues underwent gradual functional maturation and exited from the cell cycle with time of culture. By the use of an inducible expression system, we further demonstrated that the duration of caBRAF expression was the determinant of the severity and reversibility of the observed proliferative and functional changes. Collectively, we showed that a targeted increase in ERK activity in neonatal CMs can induce a progrowth, immature cell phenotype, studies of which may lead to new therapies for congenital cardiomyopathies and heart regeneration.

Our immunostaining and gene expression analyses were suggestive of both altered ECM composition and cytoskeletal changes contributing to caBRAF tissue remodeling and increased stiffness. In the context of cancer progression, a stiffer extracellular environment induced by RAS–receptor tyrosine kinase oncogene–expressing cells was necessary to amplify mechanotransduction-induced, Yes1 associated transcriptional regulator/Tafazzin (YAP/TAZ)-dependent tumorigenic cell reprogramming and proliferative growth (*64*). A parallel can be drawn between these studies and our observations that by 1 week of culture, the stiffness of caBRAF ECTs was increased while CM cycling remained unchanged and that by 2 weeks and beyond, continued tissue stiffening was accompanied by increased cell cycling in both CMs and fibroblasts (fig. S5). However, brief dox-inducible caBRAF expression was able to increase cell cycle activity without changing ECT stiffness (fig. S12), suggesting that tissue stiffening is not required for the observed proproliferative effects. While modest cell and tissue stiffening are characteristic of natural ECT maturation in vitro and postnatal cardiac development in vivo (*65*), they are associated with progressive cell cycle exit rather than sustained proliferation. Thus, the temporal dynamics of caBRAF-induced ERK signaling during 4-week NRVM ECT culture may be qualitatively different from the ERK dynamics during postnatal heart development in vivo (*17*, *51*, *66*) and will require further studies.

In addition to increased ECT stiffness and decreased contractile strength, we consistently found that caBRAF expression drove slower contraction kinetics in a primarily CM-autonomous manner (figs. S1, S3, and S5), with soluble paracrine factors being a minor contributor to this phenotype (Fig. 3G). In CMs, twitch rise and decay time are significantly influenced by the rate of calcium release from the sarcoplasmic reticulum via RYR2 receptors and uptake into the sarcoplasmic reticulum via the SERCA pump (*67*). Both RYR2 and SERCA (*Atp2a2*) were down-regulated in caBRAF ECTs, among other calcium handling genes (fig. S6), which could contribute to weaker and slower contraction. In addition, it is possible that sarcomere loss contributed not only to a decrease in twitch amplitude but also to slowed twitch kinetics (*68*), although a moderate increase in twitch decay time could be induced by ERK activation in the absence of sarcomere disorganization (Figs. 3 and 4 and fig. S11). It is interesting that functional deterioration in caBRAF ECTs was mainly observed at culture week 2 and onward (fig. S5), while transcriptomic differences were already relatively stable from culture week 1 (fig. S6). We therefore suspect posttranscriptional regulation of contractile and metabolic genes to be different between early and later (>1 week) stages of in vitro culture, potentially due to changes in cellular age or environment. This is supported by the observation that force amplitude of ECTs was decreased even if caBRAF expression was only briefly induced at later culture times (figs. S12 and S13).

Considering ERK is commonly implicated in cardiac hypertrophy (*6*, *7*, *51*), we were intrigued to not observe significantly enlarged size of caBRAF-expressing CMs in flow cytometry analysis of 2D cultures (Fig. 2E) or a prominent hypertrophic signature in RNA sequencing analysis of 3D ECTs. Instead, the larger size of caBRAF ECTs appeared to be induced by increased numbers of cells, especially at the later time points in culture. The cell growth within ECTs may have been in part limited by the lack of vasculature and reliance on the diffusion for oxygen and nutrient supply, which likely led to the development of a central necrotic core (*41*) observed in caBRAF ECTs (Fig. 1, B and E, and fig. S3, A and D). In addition, despite a difference in stiffness, both 2D and 3D culture environments led to substantial sarcomere disassembly in caBRAF-expressing CMs. While the relatively soft ECTs could attenuate endogenous ERK signaling at the level of membrane receptors (*69*), the lentivirally delivered constitutively active BRAF-driven intracellular signaling transmission and ERK activation were likely stiffness and environment independent. Sarcomere disassembly was also observed in mouse hearts in vivo upon transient activation of caERBB2 and ERK in CMs (*23*, *57*); however, this phenotype was associated with CM hypertrophy and AKT coactivation, which was absent in caBRAF ECTs (Fig. 4A). AKT activation and cardiac hypertrophy were also observed in transgenic mice expressing *BRAF-V600E* from the endogenous locus, while ERK activity remained unaffected (*16*). Comparing these results with our own suggests that different degrees of AKT and ERK activation may underlie hypertrophic/hyperplastic decisions in CMs. It thus remains to be studied whether the complex interplay between MAPK/ERK and phosphatidylinositol 3-kinase/AKT pathway (*63*, *70*, *71*) is differently regulated at different stages of CM development and by different methods of MAPK manipulation. Related, RASopathy patients with inherited gain-of-function mutations in MAPK pathway typically present with hypertrophic cardiomyopathy (*26*, *72*–*74*), but similar to our studies, CMs in some of these patients are hyperplastic rather than hypertrophied (*25*, *27*). Since different mutations in the same MAPK gene can result in differential effects on p-ERK and p-AKT levels (*23*, *24*, *41*, *75*, *76*), detailed genotype-phenotype studies will be required to understand and potentially alleviate cardiac pathology in these diseases.

While a metabolic switch from glycolysis to oxidative phosphorylation is a hallmark of CM maturation (*77*, *78*), reversion back to glycolysis has been suggested as an important step in inducing CM proliferation and heart regeneration in vivo (*52*, *79*, *80*). Notably, in tumorigenesis, ERK activation has been shown to promote metabolic reprogramming favoring glycolysis as a means to outcompete neighboring cells for energy (*81*). ERK activation via caBRAF expression in NRVM ECTs mirrored that process, as many significantly down-regulated GO terms indicated disrupted aerobic metabolism (Fig. 2D), while follow-up studies of ECT-conditioned medium confirmed near-exhaustion of glucose and significant lactate accumulation (Fig. 2G). Together, similar to studies in mouse and zebrafish hearts (*52*), ERK activation in NRVMs within ECTs stimulated glycolysis supportive of CM proliferation and tissue growth.

Use of the small-molecule inhibitor SCH772984 (Fig. 4) in our studies showed that ERK inhibition was sufficient to prevent, but not reverse, most of the caBRAF-induced phenotype. Thus, while ERK activation is required for the early phase of caBRAF-induced phenotypic changes, once established, the phenotype is maintained in a largely ERK-independent manner aside from S phase entry that still appears ERK dependent. These results imply that additional factors are required to mature the CMs after caBRAF expression rather than just removal of the caBRAF/ERK stimulus. This contrasts what has been reported in adult mice, where transient caERBB2-induced CM dedifferentiation was followed by spontaneous redifferentiation (*23*). Further studies of what mechanisms underlie these in vivo versus in vitro differences could help identify factors effective in promoting CM maturation in vitro.

Our experiments with dox-inducible caBRAF expression revealed that increased CM cycling and functional decline occurred rapidly following dox treatment, requiring as little as 2 and 4 days of dox, respectively, to observe the phenotypes (fig. S12). With longer caBRAF expression (6-day dox; Fig. 5 and fig. S9), the functional deficit and sarcomere disassembly persisted even after a ~12-day dox washout (for day 2 to day 8 dox), despite normalization of S phase entry and p-ERK abundance. As sarcomere disassembly would be expected to facilitate CM mitosis (*82*, *83*), these results further supported requisite roles of ERK activation for NRVM cycling in ECTs. On the other hand, the moderate functional decline following 2-day dox treatment (fig. S10D) was fully reversible (Fig. 5H), and, unexpectedly, it was associated with reduced EdU incorporation (Fig. 5I), possibly due to continued negative feedback from up-regulated MAPK and cell cycle inhibitory proteins (dual specificity phosphatase 4/5/6 (DUSP4/5/6) and cyclin dependent kinase inhibitor 2B (CDKN2B); all elevated in our RNA sequencing dataset; fig. S6A). Since the duration of ERK activation has been shown to influence resulting changes in gene expression (*12*), future studies using the dox-inducible system are warranted to gain mechanistic insights into these findings.

Our study is limited by the use of NRVMs, which are neonatal, making them more likely to undergo proliferation than adult CMs. However, NRVMs have relatively advanced maturation and lower proliferation rates than hiPSC-CMs (*42*, *48*, *84*), and, thus, NRVM ECTs are a more stringent in vitro system to study cardiac mitogens compared to human ECTs (*41*, *85*–*87*). Considering that our measurements focused on EdU incorporation, phosphorylated histone H3 expression, and nuclear counts, we can only conclude that caBRAF expression increased rates of CM cell cycle entry and mitosis/karyokinesis, rather than inducing definite cytokinesis. Since soluble factors did not significantly contribute to the observed phenotype (Fig. 3 and fig. S8), future studies are warranted to examine how CM-specific caBRAF expression alters juxtacrine and matrix-mediated signals. Further studies of the effects of caBRAF expression in vivo will be necessary to determine how our observations in ECTs made from neonatal CMs relate to the developing or adult heart environment. For this purpose, a slower-expressing nonintegrating adeno-associated virus should be applied rather than integrating LVs that we used in this in vitro study to ensure rapid expression kinetics. Last, while we focused on the most common BRAF mutant, *BRAF-V600E*, other BRAF variants that also activate ERK (*13*, *28*), may confer distinct phenotypes on CMs based on specific ERK activation levels.

In summary, we have shown that *BRAF-V600E*–mediated ERK activation in NRVMs can induce sustained or reversible changes in cell cycling, function, glycolysis, and migratory capacity. In many cell types including CMs, ERK is known to play an integral role in intracellular signaling response to environmental cues (*18*, *88*). Considering the elaborate negative feedback that regulates MAPK overactivation (*11*), our study contributes to the growing understanding of how targeting of specific steps in this pathway can direct type and persistence of resulting cell response. In the future, methods to manipulate MAPK signaling in CMs may lead to cardiac reparative strategies in pediatric or adult patients; however, precise control of the pathway will be critical to avoid irreversible detrimental effects on the CM phenotype.

## MATERIALS AND METHODS

### NRVM isolation and 2D culture

All animal procedures were performed in compliance with the Institutional Animal Care and Use Committee at Duke University and the NIH Guide for the Care and Use of Laboratory Animals (Institutional Animal Care and Use Committee (IACUC) reference A064-21-03). NRVMs were isolated as previously described (*42*, *43*, *84*). Briefly, ventricles were harvested from postnatal day 2 male and female Sprague-Dawley rat pups, minced finely, and pooled before overnight trypsin incubation at 4°C. The following day, the minced ventricular tissue was subjected to several collagenase digestion and filtering steps to yield single-cell suspension. Cells were preplated for 1 hour to remove nonmyocytes and enrich the NRVM population. The nonadherent cells were resuspended in 2D cardiac medium [low-glucose Dulbecco’s modified Eagle’s medium (DMEM), 10% fetal bovine serum (FBS), penicillin (5 U/ml), and vitamin B12 (2 μg/ml)] and plated onto fibronectin-coated Aclar coverslips at a density of 5 × 10^5^ cells per well of a 12-well plate. Twenty-four hours following plating the medium was changed to a reduced serum medium [low-glucose DMEM, 5% FBS, penicillin (5 U/ml), and vitamin B12 (2 μg/ml)], and full medium changes were performed every other day. In experiments involving additions of recombinant OPG (5 to 125 ng/ml) or GDF-15 (1 to 25 ng/ml), medium containing the recombinant protein was changed daily.

### ECT fabrication and 3D culture

NRVM ECTs were prepared as previously described (*42*–*44*). Briefly, 6.5 × 10^5^ freshly isolated NRVMs were mixed with a fibrin-based hydrogel [fibrinogen (2.5 mg/ml), thrombin (1 U/ml), and 10% (v/v) Matrigel] and cast in polydimethylsiloxane tissue molds with two 2-mm by 7-mm troughs and a porous nylon frame. The molds containing the hydrogel-cell mixture were incubated at 37°C for 45 min to allow the hydrogel to fully polymerize and attach to the nylon frame. Tissues were then immersed in 3D cardiac medium [low-glucose DMEM, 10% horse serum, 0.5% chick embryo extract, aminocaproic acid (1 mg/ml), ascorbic acid 2-phosphate sesquimagnesium salt hydrate (50 μg/ml), penicillin (5 U/ml), and vitamin B12 (2 μg/ml)]. The following day, the ECTs on frames were carefully removed from the molds and cultured under dynamic conditions on a rocker. Full medium changes of 2 ml per well were performed every other day for the duration of the experiment. For coculture experiments, a nontransduced ECT was cultured with another ECT transduced with either Ctrl LV or caBRAF LV, and the nontransduced ECT was analyzed. Dox was used in indicated experiments at a concentration of 2 μg/ml and was readministered every other day.

### Migration assay in 2D NRVM cultures

For assaying cell migration, mitomycin C was added the day after seeding to inhibit fibroblast proliferation and ensure assessment of predominantly cardiomyocytes. The cells (1 week after seeding) were time lapse–imaged on a Dragonfly spinning disk confocal microscope (Andor), taking one image every 10 min for ~6 hours while maintaining CO_2_ and temperature.

### Small-molecule ERKi experiments

For experiments using small-molecule inhibitor of ERK, SCH772984 (ERKi) was used. The inhibitor was applied from days 8 to 14 or 2 to 14 after tissue generation at 100 nM, and ERKi-treated ECTs were compared to a dimethyl sulfoxide vehicle control.

### Cloning of mitogen constructs

For generation of the mitogen construct caBRAF, plasmid containing the gene sequence was used as polymerase chain reaction template for amplification of insert before cloning. Polymerase chain reaction primers were designed to add complementary restriction site overhangs to the gene inserts that were also present in the MHCK7-MCS-P2A-mCherry backbone used for cloning the constructs. Standard restriction cloning was used to insert the gene fragments. Sanger sequencing was performed to ensure maintenance of reading frame and correct sequence.

### Preparation of LVs

LVs were prepared as previously described (*89*). Briefly, human embryonic kidney–293T cells were cultured in high-glucose DMEM containing 10% FBS and 1% penicillin/streptomycin. Plasmids (construct plasmid, Pax2, and vesicular stomatitis virus glycoprotein) were purified using midiprep before transfection into human embryonic kidney–293T cells at 65 to 75% confluence using Jetprime transfection reagent. Medium was changed 16 hours following transfection, and medium containing virus was harvested 3 to 4 days following initial transfection. Virus was purified by precipitation using 3 volumes of medium to 1 volume of 40% PEG-8000 at 4°C overnight and then pelleted by centrifugation at 1500*g* for 45 min at 4°C. Precipitated virus was aliquoted and stored at −80°C before use. For NRVM monolayer experiments, viral suspension was added at the time of cell plating. For NRVM ECT experiments, LVs were added to the hydrogel-cell mixture at the time of ECT fabrication. All LV doses were titrated to yield transduction efficiency between 60 and 80% based on fluorescent protein expression. On the basis of viral tittering using postnatal day 24 enzyme-linked immunosorbent assay (Takara Bio), multiplicity of infection was 4 for MHCK7::caBRAF-mCherry, 1 for the dox-inducible construct, and 1 for the control LV.

### Flow cytometry

NRVM monolayers were rinsed with phosphate-buffered saline (PBS) and then dissociated using 0.05% Trypsin-EDTA at 37°C for 3 min, upon which monolayers were triturated several times to yield a single-cell suspension. Trypsin was quenched with DMEM/F12 containing 20% FBS and deoxyribonuclease I (20 μg/ml). The cell suspension was centrifuged at 300*g* for 5 min, then resuspended in 4% paraformaldehyde (PFA) diluted in PBS. Cells were incubated in PFA for 10 min at room temperature, centrifuged again, and then resuspended in PBS containing 5% FBS for storage at 4°C.

Cells were stained for flow cytometry after centrifugation at 300*g* for 5 min to remove storage medium. If EdU staining was performed, then cells were incubated with the EdU flow cytometry staining cocktail as per the manufacturer’s protocol (Thermo Fisher Scientific) and incubated in the dark for 30 min, and then washed 2× by addition of PBS, followed by centrifugation. Antibody staining was performed after EdU staining. For antibody staining, cells were resuspended in fluorescence-activated cell sorting (FACS) buffer [PBS with 0.5% bovine serum albumin (BSA), 0.1% Triton X-100, and 0.02% sodium azide]. Primary antibodies including an isotype control were diluted in FACS buffer and incubated for 1 hour on ice. Cells were washed two times with FACS buffer before addition of secondary antibodies and Hoechst diluted in FACS buffer. Secondary antibodies were incubated for 30 min at room temperature. Samples were run on a BD LSRFortessa X-20.

### Immunostaining and imaging

Performed as previously described (*41*), cell monolayers were fixed with 4% (v/v) PFA at room temperature for 15 min, then blocked in antibody buffer [5 w/v donkey serum and 0.1% (v/v) Triton X-100 in PBS] for 30 min at room temperature, and incubated with primary antibodies for 30 min in antibody buffer. Primary antibody sources and dilutions are indicated in the Key Resources Table. The monolayers were washed with PBS before incubation with Alexa Fluor–conjugated secondary antibodies at 1:1000 and Hoechst at 1:200 in antibody buffer for 30 min. Monolayer samples were mounted using Fluoromount-G mounting medium and imaged using an Andor Dragonfly spinning disk confocal microscope. All antibodies and their respective dilutions used in this manuscript are in table S1.

ECTs were fixed with 2% (v/v) PFA on a rocking platform at 4°C overnight. For cross-sectional analysis, the fixed tissues were suspended in optimal cutting temperature compound (OCT) and flash-frozen in liquid nitrogen until solidified. The frozen tissue blocks were sectioned using a cryostat (Leica) into 10-μm sections. ECT cross sections were blocked in antibody buffer for 2 hours at room temperature. Whole tissues for longitudinal images were blocked overnight at 4°C. All samples were incubated with primary antibodies 4°C overnight in antibody buffer. Primary antibodies were used at the indicated dilutions in the Key Resources Table. Samples were incubated with Alexa Fluor–conjugated secondary antibodies at 1:1000 and Hoechst at 1:200 in antibody buffer for 2.5 hours at room temperature for cross sections and overnight at 4°C for whole tissues. Cross sections and unsectioned whole ECTs were mounted with hard-set mounting medium (Antifade Glass) and imaged using an Andor Dragonfly spinning disk confocal microscope.

### RNA sequencing

ECTs were pooled (six to eight per sample, each sample was from a separate NRVM isolation from a total of three isolations) and homogenized in RLT buffer (QIAGEN) using green RINO homogenization tubes following the manufacturer’s recommendations. RNeasy Fibrous Tissue Mini Kit (QIAGEN) was used to isolate RNA from the homogenized tissue, which was then sent for RNA sequencing by Genewiz. Genewiz performed library preparation and sequencing; briefly, ribosomal RNA was removed using polyadenylate selection for mRNA species, and sequencing was performed using Illumina HiSeq, 2 × 150–base pair paired-end reads with 20 to 30 million reads per sample. Read quality was confirmed using FastP software (*90*, *91*). Alignment to the *Rattus norvegicus* genome assembly was performed using the Rsubread package (*92*). Differential expression was performed using DESeq2 (*93*). Significantly differentially expressed genes were classified as follows: absolute value of log_2_FC (log_2_ fold change) > 1.5 and *P*_adj_ < 0.01.

### Western blot

To isolate total protein from ECTs, cells were rinsed twice with ice-cold PBS before lysis with radioimmunoprecipitation assay buffer containing protease inhibitor cocktail (Sigma-Aldrich, P8340) and phosphatase inhibitor cocktail 3 (Sigma-Aldrich, P0044). Cells were incubated on ice for 10 min, and then lysates were collected and spun down at 10,000*g* to pellet debris. Supernatants were measured using a bicinchoninic acid (BCA) assay to determine total protein concentration. Thirty micrograms of each sample was run on a 4 to 12% gradient gel with tris-glycine-SDS running buffer at 100 V for 1 to 1.5 hours depending on the size of proteins being separated. Proteins were transferred to 0.45-μm polyvinylidene difluoride membranes for Western blot at 4°C at 60 V for 2 hours. Membranes were blocked overnight in 3% BSA in tris-buffered saline (TBS). Membranes were cut such that multiple size proteins could be blotted from the same membranes. Primary antibodies were diluted in 3% BSA and incubated with membranes overnight at 4°C. Membranes were washed 3× with TBS containing 0.1% Tween 20 (TBS-T) before incubation with horseradish peroxidase–conjugated secondary antibodies. Membranes were washed 3× with TBS-T before incubation in SuperSignal West Pico PLUS Chemiluminescent Substrate for 5 min. Membranes were imaged with a Bio-Rad ChemiDoc using signal accumulation mode for up to 2 min. If an additional protein of interest was similar in size to the housekeeping gene (LamB1) or other proteins of interest, then membranes were stripped following exposure for 10 min using Restore PLUS Western blot Stripping Buffer. Membranes were then reblocked and reprobed as indicated above. All antibodies and their respective dilutions used in this manuscript are in table S1.

### Conditioned medium assays

For the cytokine array, R&D Systems’ Proteome Profiler Rat XL Cytokine Array was used on the basis of the manufacturer’s recommendations. Membranes were imaged with a Bio-Rad ChemiDoc using signal accumulation mode for up to 2 min. To measure the concentration of glucose in the collected culture medium, the Amplex Red glucose assay kit from Invitrogen was used following the manufacturer’s recommendations. To measure the concentration of lactate in the collected culture medium, we performed a plate reader assay as we have shown previously (*94*). Briefly, a lactate dehydrogenase–catalyzed reaction with lactate, nicotinamide adenine dinucleotide (oxidized form), and hydrazine was used, and the formation of reduced nicotinamide adenine dinucleotide was measured spectrophotometrically at 340 nm kinetically up to 60 min. The concentration of lactate in the medium was then calculated on the basis of the linear range of a standard curve.

### Force measurements

ECT force generation was measured using a custom-made force measurement setup consisting of an optical force transducer and linear actuator as previously described (*36*, *42*, *43*, *48*, *95*). In 37°C Tyrode’s solution, the ECT was pinned to chamber at one end and a polydimethylsiloxane float connected to a linear actuator controlled by LabVIEW software at the other end. Using platinum electrodes, a 90-V biphasic electrical pulse was applied for 5 ms at 2-Hz rate to induce contractions. The force measurements were performed at the ends of 4% stretch steps lasting 45 s until 12% stretch was reached. Stretch distance to achieve 4% stretch steps was determined on the basis of the 7-mm resting length for control tissues. Because of high-tissue stiffness with caBRAF, 0% stretch was measured starting at the point of zero passive force, which could be less than 7 mm. Maximum twitch amplitude (occurring anywhere between 0 and 12% stretch), passive force–length curves, and parameters of twitch kinetics were derived as previously described using custom MATLAB software (*96*).

### Optical mapping of action potential propagation

Action potential propagation in NRVM ECTs was assessed using optical mapping, as previously described (*36*). Briefly, tissues were stained with 10 μM Di-4-ANEPPS for 6 min with 10 μM blebbistatin to prevent motion artifacts and transferred to a 37°C recording chamber filled with Tyrode’s solution. Action potential propagation was initiated by 2-Hz stimuli from a bipolar point electrode, and the signals were collected by a 19-mm-diameter optical fiber array through a custom 3:1 fiber optic taper. Isochrone map construction and conduction velocity and action potential duration calculations were performed using custom MATLAB software, as previously described (*97*).

### Image analysis

Image analysis was performed using custom Fiji (*98*) macros. Colocalization analysis between vimentin signal and nuclei as well as EdU signal and nuclei was performed to exclude proliferative fibroblasts from cardiomyocyte EdU quantification. A custom Fiji macro using autothresholding methods was used to determine ECT F-actin^+^ area and total CSA.

### Migration analysis

Cell migration analysis on time-lapse images was performed using the TrackObjects module in CellProfiler (*99*) to determine cell position, root mean square displacement, and cell speed. The resulting cell speed and position data were fit to a Persistent Random Walk model as shown previously (*100*, *101*) using custom MATLAB code to calculate mean square displacement, persistence time, and mean free path. For mean square displacement analysis, cells tracked for less than 100 consecutive seconds were excluded from analysis. Migratory cells were considered to have a mean free path of >0.1 μm based on measurements of visually nonmigratory cells.

### Statistical analysis

Statistical analysis was performed with GraphPad Prism software. Outliers were identified and removed using GraphPad Prism 8.3.0 ROUT method (*Q* = 1%). Normality testing was done using the Shapiro-Wilk test, and testing for equal variances was done using the Brown-Forsythe test. If data were not normally distributed, we performed logarithmic transformations and retested for normality and equal variances before performing the appropriate statistical test. Analyses of variance (ANOVAs) were run with post hoc multiple comparisons testing for experiments containing multiple groups, and unpaired *t* tests were run in experiments comparing just two groups. All experiments were carried out in multiple cell batches (*N*), while a single engineered tissue or single well in a culture dish is defined as *n*.
