# Supplementary material for: Time-dependent effects of BRAF-V600E on cell cycling, metabolism, and function in engineered myocardium
Source: Sci Adv. 2024 Jan 24;10(4):eadh2598. doi: 10.1126/sciadv.adh2598 (PMC10807800; doi:10.1126/sciadv.adh2598)
Supplement: Supplementary file 1 — Figs. S1 to S14 Table S1 Legend for data file S1 [file sciadv.adh2598_sm.pdf]

Science Advances
AAAS

Supplementary Materials for

Time-dependent effects of BRAF-V600E on cell cycling, metabolism, and function in engineered myocardium

Nicholas Strash *et al.*

Corresponding author: Nenad Bursac, nenad.bursac@duke.edu

Sci. Adv. **10**, eadh2598 (2024)
DOI: 10.1126/sciadv.adh2598

The PDF file includes:

Figs. S1 to S14
Table S1
Legend for data file S1

Other Supplementary Material for this manuscript includes the following:

Data file S1

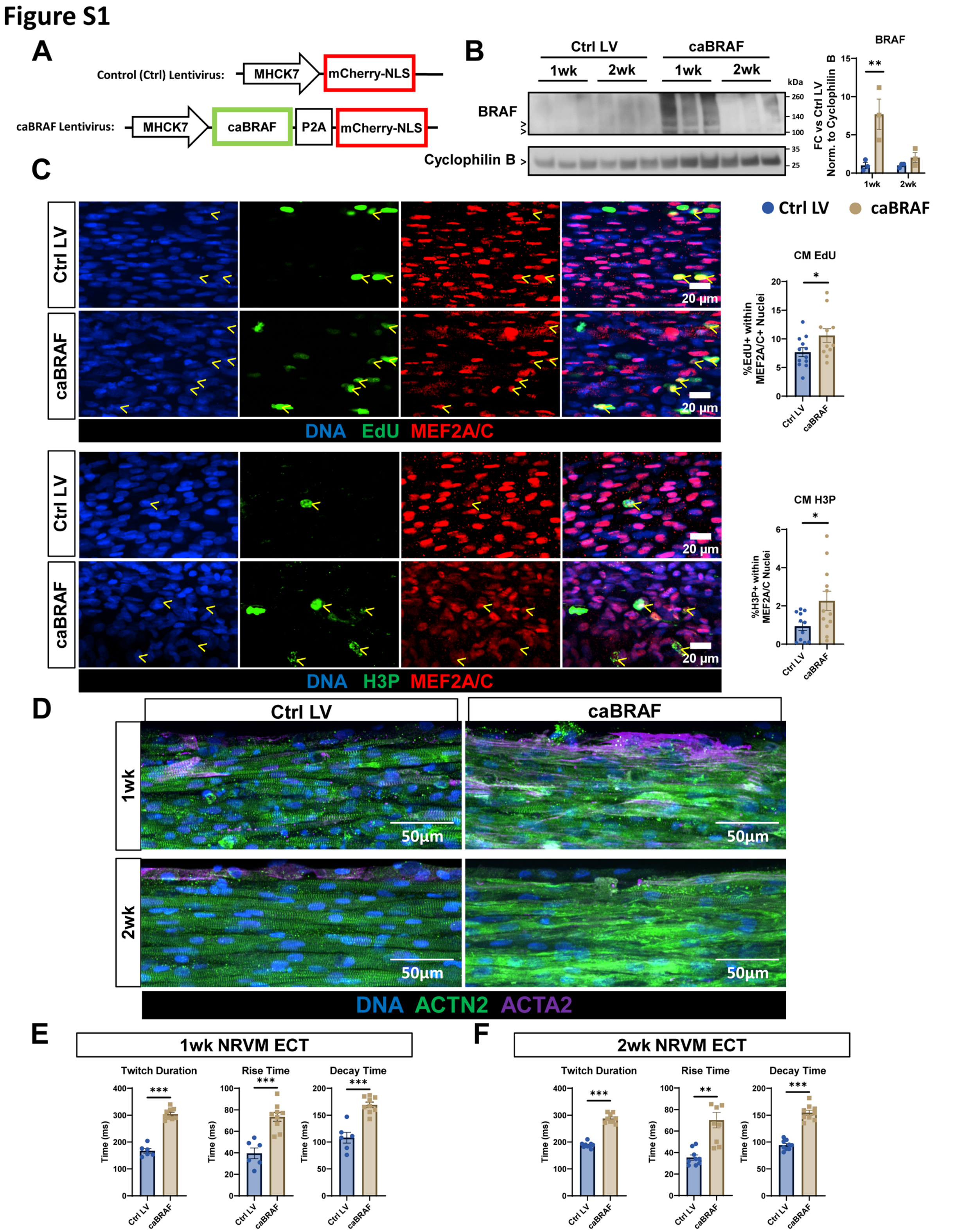

Figure S1. Lentiviral caBRAF Expression Induces CM Cycling, Sarcomeric Deterioration, and Prolonged Twitch Kinetics in NRVM ECTs. (A) Schematic of control (Ctrl) and caBRAF lentivirus (LV) constructs. (B) Western blots and corresponding quantification of BRAF

abundance normalized to Cyclophilin B in caBRAF relative to Ctrl ECTs at 1wk and 2wk of culture with arrowheads showing expected protein size. (C) Representative whole-mount images of 2wk ECTs and corresponding quantification of phosphorylated Histone H3 (H3P) and EdU incorporation in CMs (MEF2A/C+) with arrowheads indicating double-positive quantified cells. (D) Representative whole-mount images of 1 wk and 2 wk NRVM ECTs immunostained for sarcomeric α-actinin (ACTN2) and smooth muscle actin (ACTA2). (E-F) Twitch kinetics of ECTs characterized by total twitch duration, time to peak, and relaxation time after (E) 1 wk and (F) 2 wk of culture. Data: n=3, each point is n=3 ECTs pooled together (B), n=11-12 ECTs (C) n=6-9 ECTs (E-F). Column graphs showing individual data points, mean ± SEM. $^{**}p < 0.01$, $^{***}p < 0.001$ vs. Ctrl LV. All experiments were repeated in N=3 independent ECT batches.

Figure S2

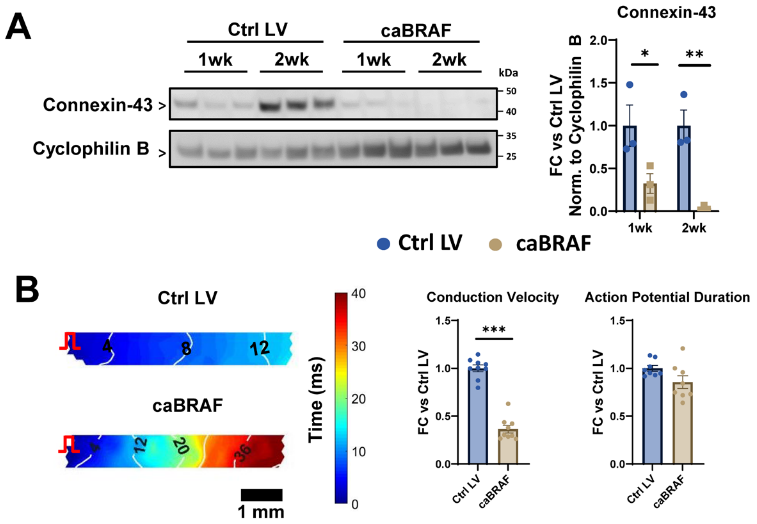

Figure S2. caBRAF Expression Reduces Connexin-43 Abundance and Slows Electrical Propagation in NRVM ECTs. (A) Western blots and corresponding quantification of BRAF abundance normalized to Cyclophilin B in caBRAF relative to Ctrl ECTs at 1wk and 2wk of culture with arrowheads showing expected protein size. (B) Representative isochrone maps of action potential conduction and corresponding quantifications of conduction velocity and action potential duration in 2wk ECTs. Data: n=3, each point is n=3 ECTs pooled together (A), n=8-9 ECTs (B). Column graphs showing individual data points, mean ± SEM. **p < 0.01, ***p < 0.001 vs. Ctrl LV. All experiments were repeated in N=3 independent ECT batches.

Figure S3

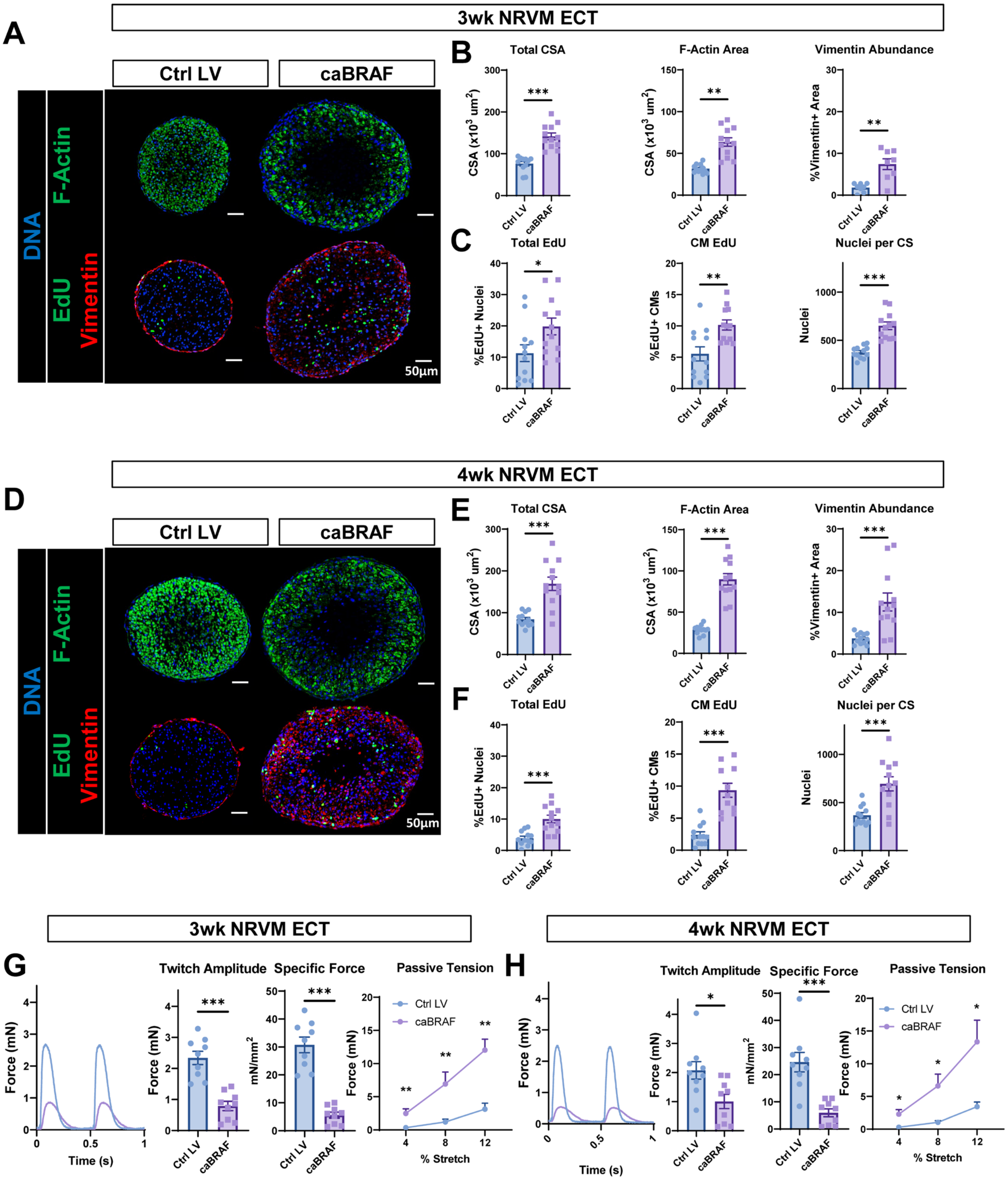

Figure S3. Sustained caBRAF Expression Induces Persistent Morphological, Cell Cycling, and Functional Changes in NRVM ECTs at 3 and 4 Weeks of Culture. (A-F) Representative images of NRVM ECT cross-sections and corresponding morphological and cell cycling quantifications after 3 wk (A-C) and 4 wk (D-F) of culture. ECT, engineered cardiac tissue; CSA, cross-sectional area. (G-H) Representative twitch force traces at 2 Hz stimulation, twitch amplitudes, specific forces (force per CSA), and passive tension-length relationships in ECTs after

3 wk (G) and 4 wk (H) of culture. %Stretch values are shown relative to the initial testing length. Data: n=11-12 ECTs (B-F), n=9 ECTs (G-H). Column graphs showing individual data points, mean ± SEM; Line plots, mean + SEM. *$p < 0.05$, **$p < 0.01$, ***$p < 0.001$ vs. Ctrl LV. All experiments were repeated in N=3 independent ECT batches.

Figure S4

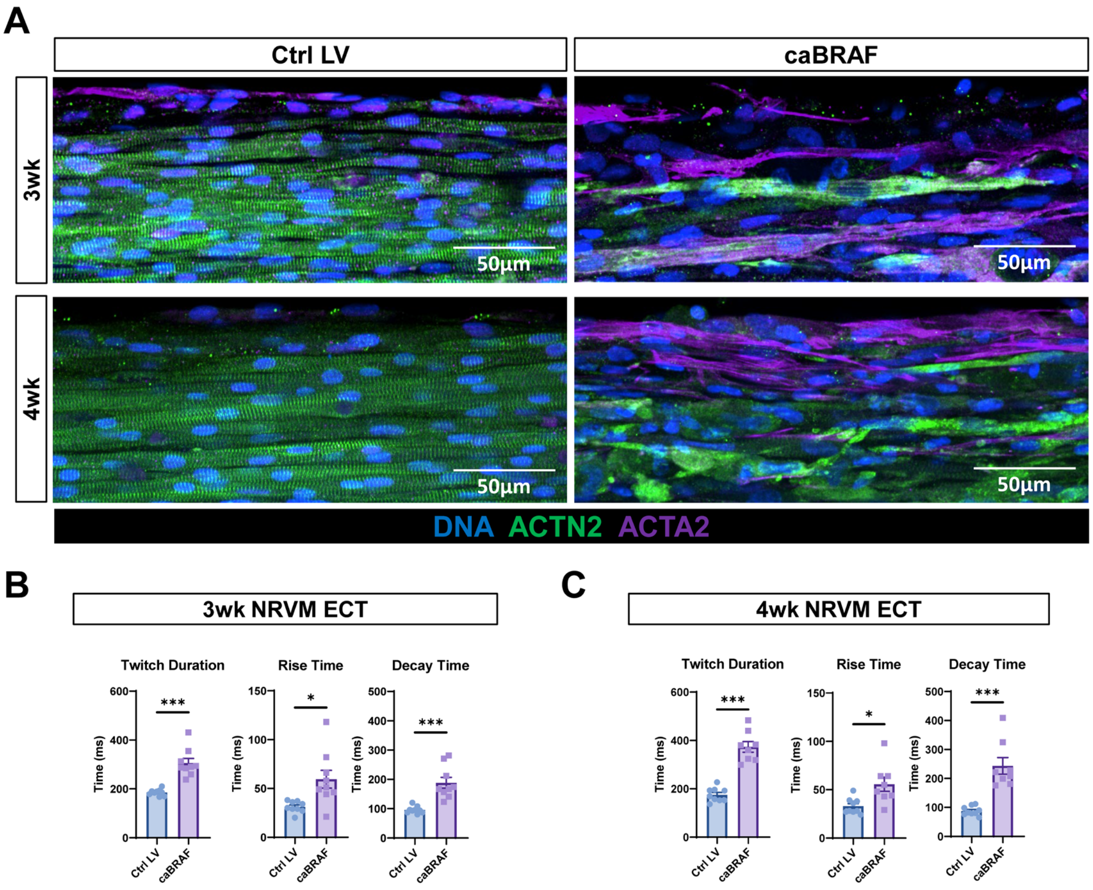

Figure S4. Sustained caBRAF Expression Induces Persistent Sarcomeric Deterioration and Slower Twitch Kinetics in NRVM ECTs. (A) Representative whole-mount images of 3 wk and 4 wk NRVM ECTs immunostained for sarcomeric α-actinin (SAA) and smooth muscle actin (SMA). (B-C) Twitch kinetics of ECTs characterized by total twitch duration, time to peak, and relaxation time after (B) 3 wk and (C) 4 wk of culture. Data: n=9 ECTs (B-C). Column graphs showing individual data points, mean ± SEM. *p < 0.05, ***p < 0.001 vs. Ctrl LV. All experiments were repeated in N=3 independent ECT batches.

Figure S5

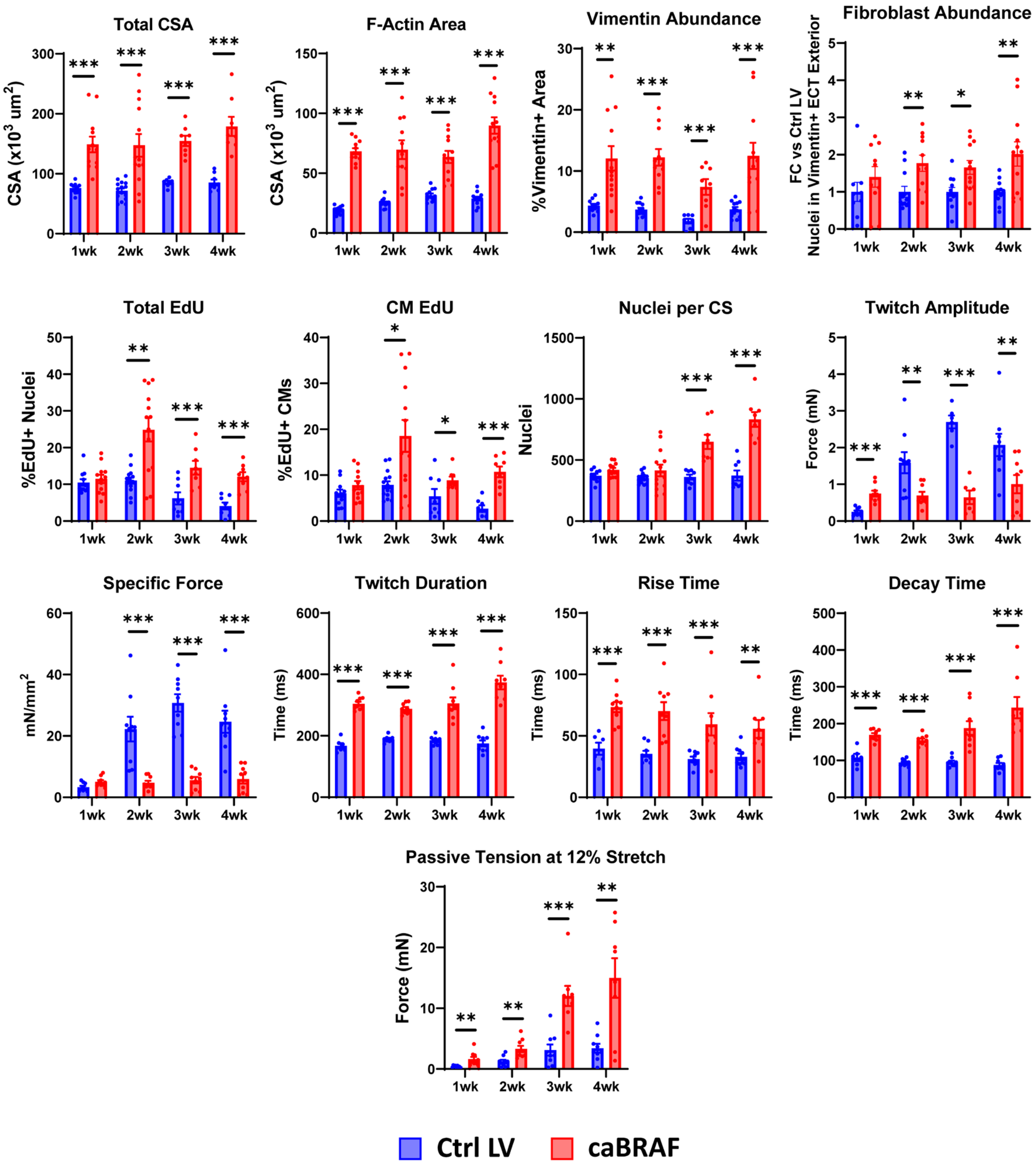

Figure S5. Compiled Morphological, Cell Cycling, and Functional Effects of caBRAF Expression in NRVM ECTs with Time of Culture. Data compiled from Figure 1 and Supplemental figures S1,3,4 showing summarized structural and functional changes due to caBRAF expression from 1 to 4 wk of culture. Data: Column graphs showing individual data points, mean ± SEM. *$p < 0.05$, **$p < 0.01$, ***$p < 0.001$ vs. Ctrl LV. All experiments were repeated in N=3 independent ECT batches.

Figure S6

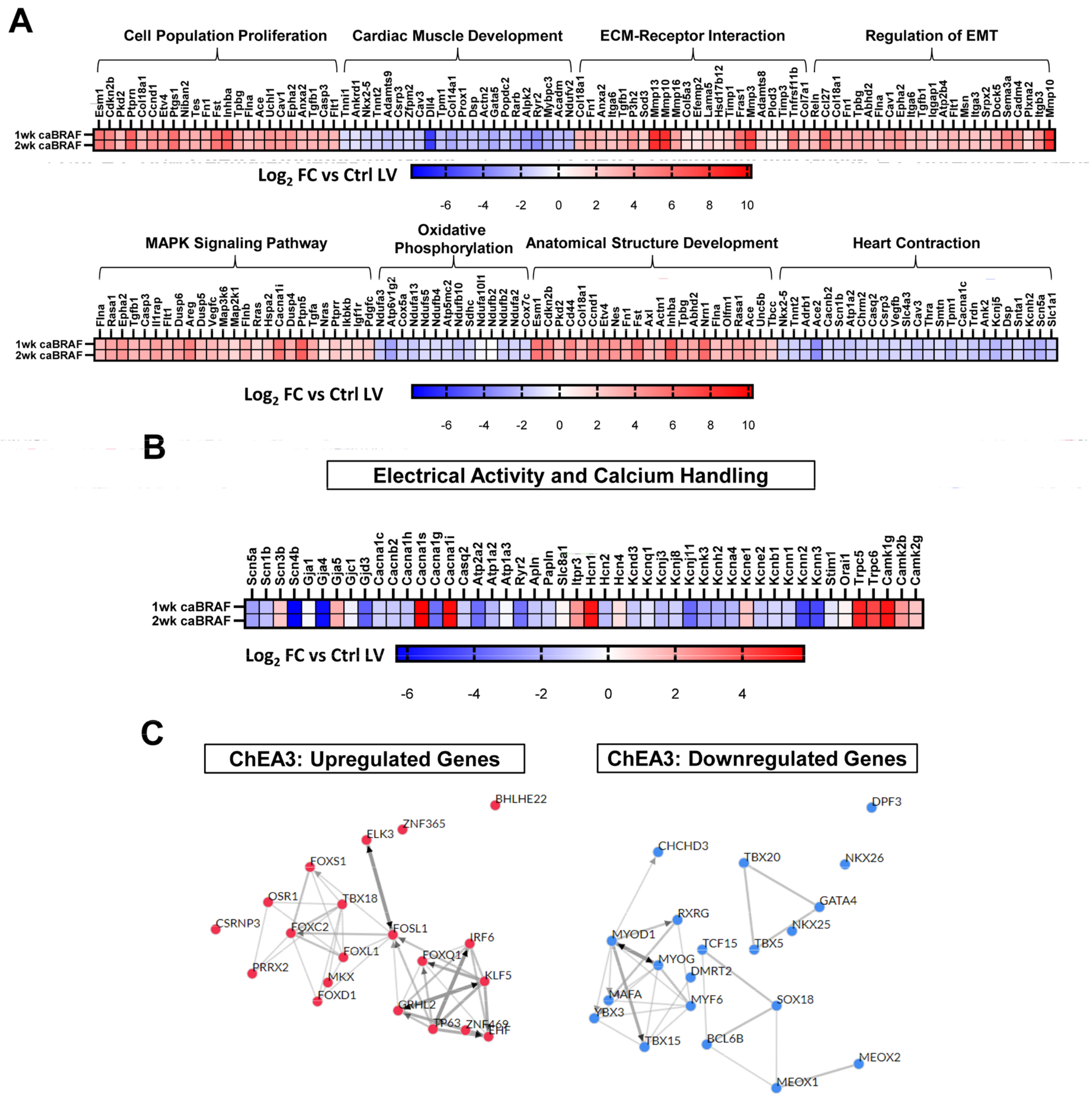

Figure S6. Heat Maps of GO Terms and ChEA3 Transcription Factor Analysis of RNA-Sequencing Data from caBRAF and Ctrl ECTs. (A) Heat maps of the top contributing genes based on adjusted p-value within the indicated GO terms. (B) Curated heat map showing genes conferring cardiac electrical activity and calcium handling. Shown in A and B are relative gene expression levels in caBRAF vs. age-matched Ctrl ECTs. (C) ChEA3 analysis showing transcription factors predicted to be responsible for observed upregulated and downregulated gene expression changes in caBRAF vs. Ctrl ECTs.

Figure S7

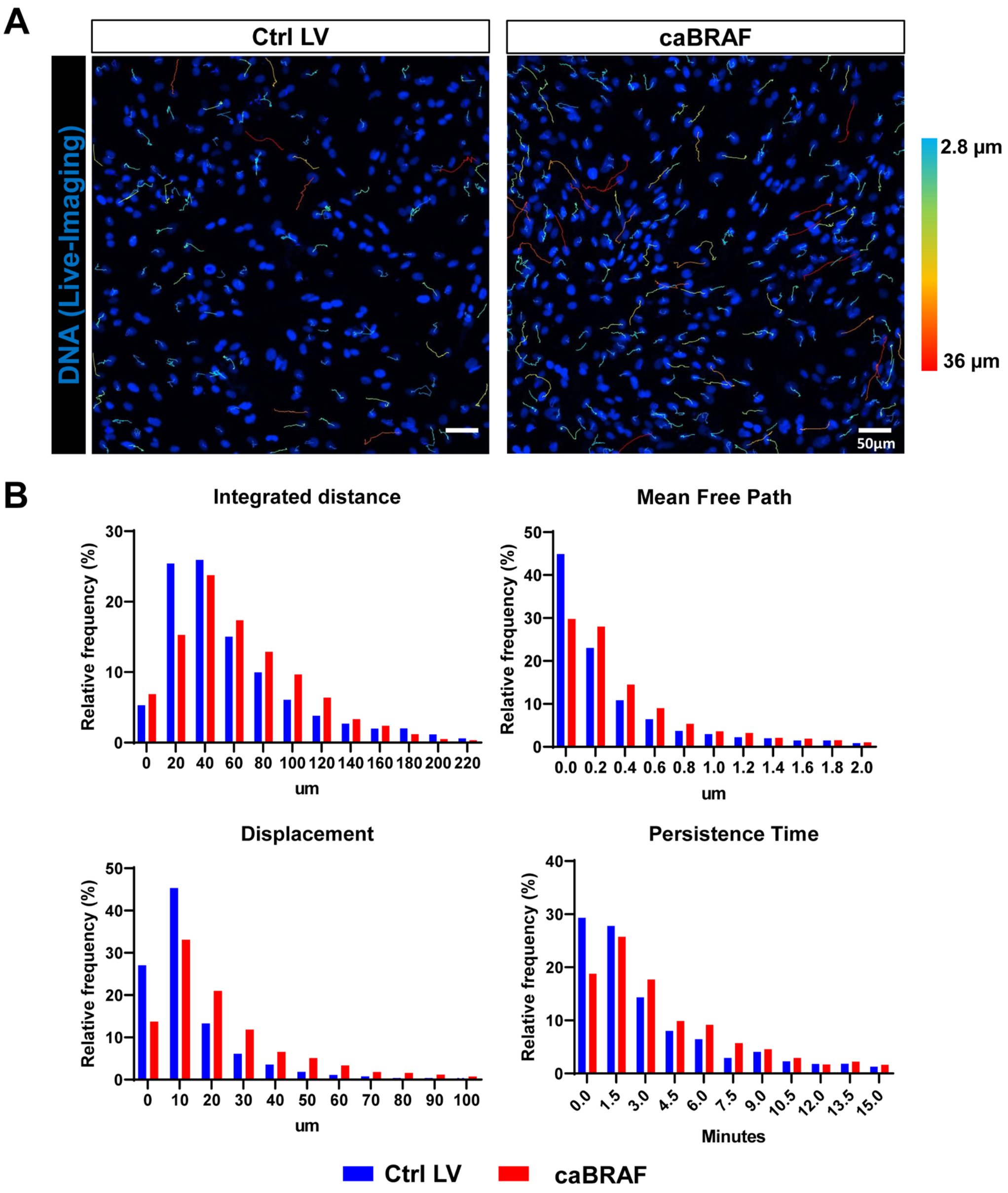

C

	Integrated Distance		*Displacement*		*Mean Free Path*		*Persistence Time*	
	Ctrl LV	caBRAF	Ctrl LV	caBRAF	Ctrl LV	caBRAF	Ctrl LV	caBRAF
Skewness	1.63	1.074	3.108	2.336	23.66	14.49	19.9	9.977
Kurtosis	2.961	1.507	13.79	7.248	669.8	231.4	506.2	123

Figure S7. Effects of CaBRAF Expression on NRVM Migratory Activity in 2D Culture. (A) Representative snapshots of live-imaged NRVMs showing superimposed color-coded nuclear paths travelled during a 6h imaging window. Live nuclei were labelled by Hoechst dye. (B-C) Population histograms of migration parameters (B) and corresponding descriptive statistics (C) showing right-shifted distribution for caBRAF NRVMs. Data: Histograms with bins centered on indicated values.

Figure S8

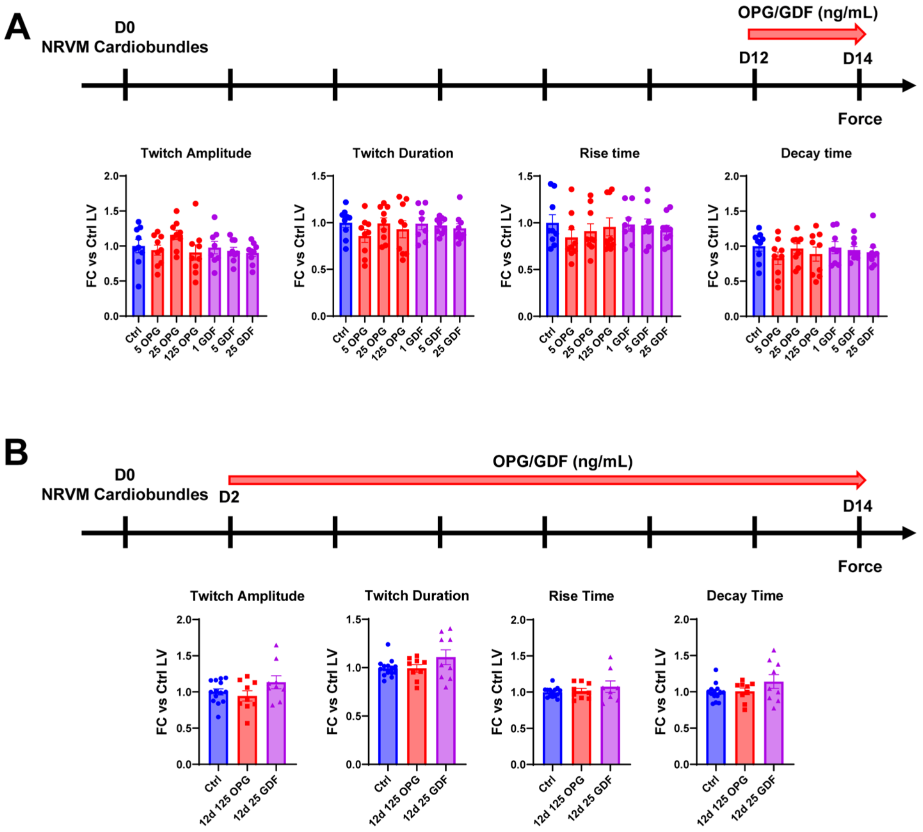

Figure S8. Supplementation of ECTs with Recombinant OPG or GDF-15 Has no Effect on Tissue Contractility. (A-B) Schematics of experimental design and quantification of contractile function in ECTs treated with different doses (in ng/mL) of OPG or GDF-15 (GDF) daily for 48hr (A) or at the highest dose during the last 12d of culture (B). Data: n=8-9 ECTs (A) n=9-14 ECTs (B). Column graphs showing individual data points, mean ± SEM. $^{*}p < 0.05$, $^{**}p < 0.01$, $^{***}p < 0.001$ vs. caBRAF+Veh. All experiments were repeated in N=3-5 independent ECT batches.

Figure S9

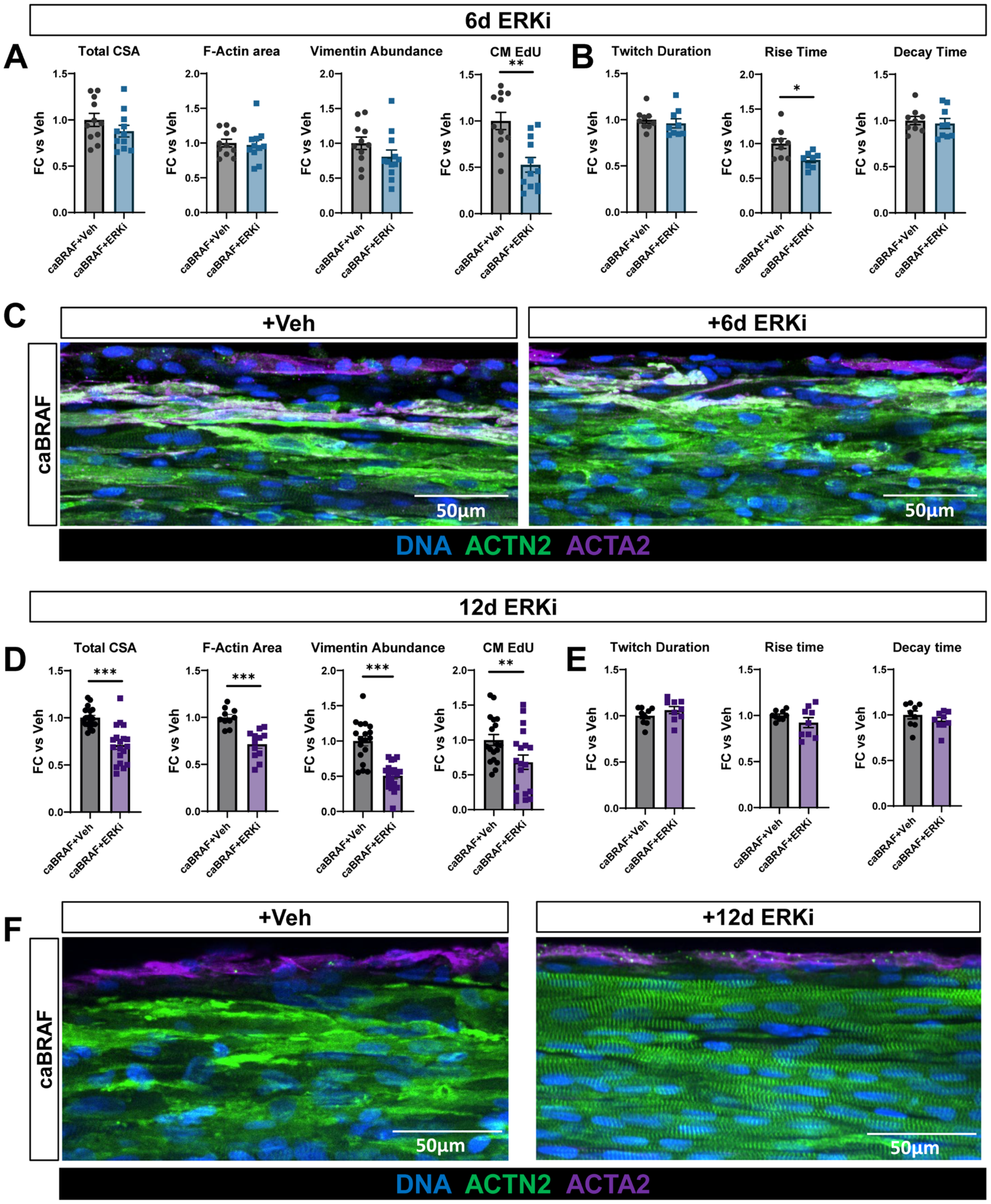

Figure S9. Long-term ERK Inhibition Prevents Sarcomere Disassembly and Morphological Changes Induced by caBRAF Expression. (A-B, D-E) Additional quantifications of (A,D) tissue morphology and cell cycling and (B,E) twitch kinetics in ECTs treated with 100nM Erki from (A-B) d8-14 or (D-E) d2-14. (C,F) Representative whole-mount images showing sarcomere structure of NRVM ECTs treated with 100nM Erki from (C) d8-14 or (F) d2-14. Data: n=11-12 ECTs (A) n=9 ECTs (B,E), n=18-19 ECTs (D). Column graphs showing individual data points, mean ± SEM. *$p < 0.05$, **$p < 0.01$, ***$p < 0.001$ vs. caBRAF+Veh. All experiments were repeated in N=3-5 independent ECT batches.

Figure S10

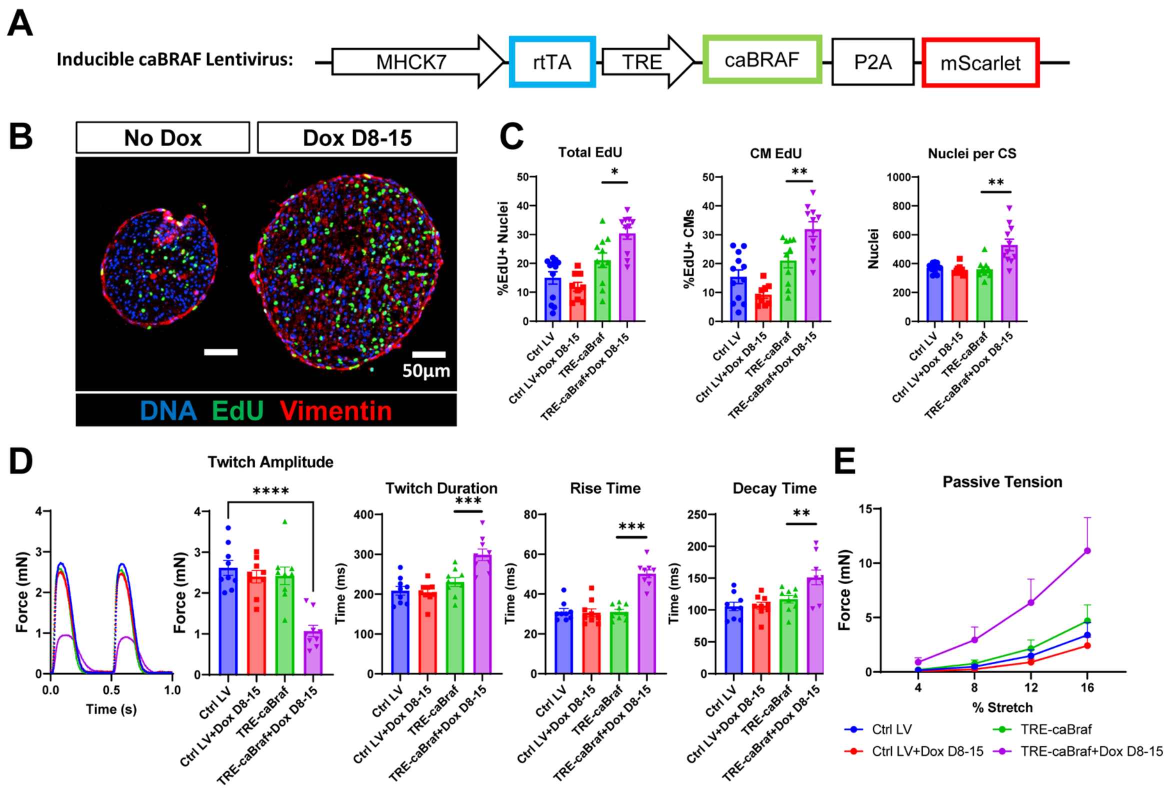

Figure S10. Establishment and Testing of a Single-vector Doxycycline-Inducible caBRAF Lentivirus in ECTs. (A) Schematic of experimental design for (B-E). (B-E) Representative cross-section images of ECTs (B) and quantifications of cell cycle parameters (C), twitch amplitude and kinetics with representative force traces (D), and passive tension-length relationships (E). Data: n=9 ECTs (C,E), n=11-12 ECTs (D). Column graphs showing individual data points, mean ± SEM. Line plots, mean + SEM. *p < 0.05, **p < 0.01, ***p < 0.001 vs. indicated group. All experiments were repeated in N=3 independent ECT batches.

Figure S11

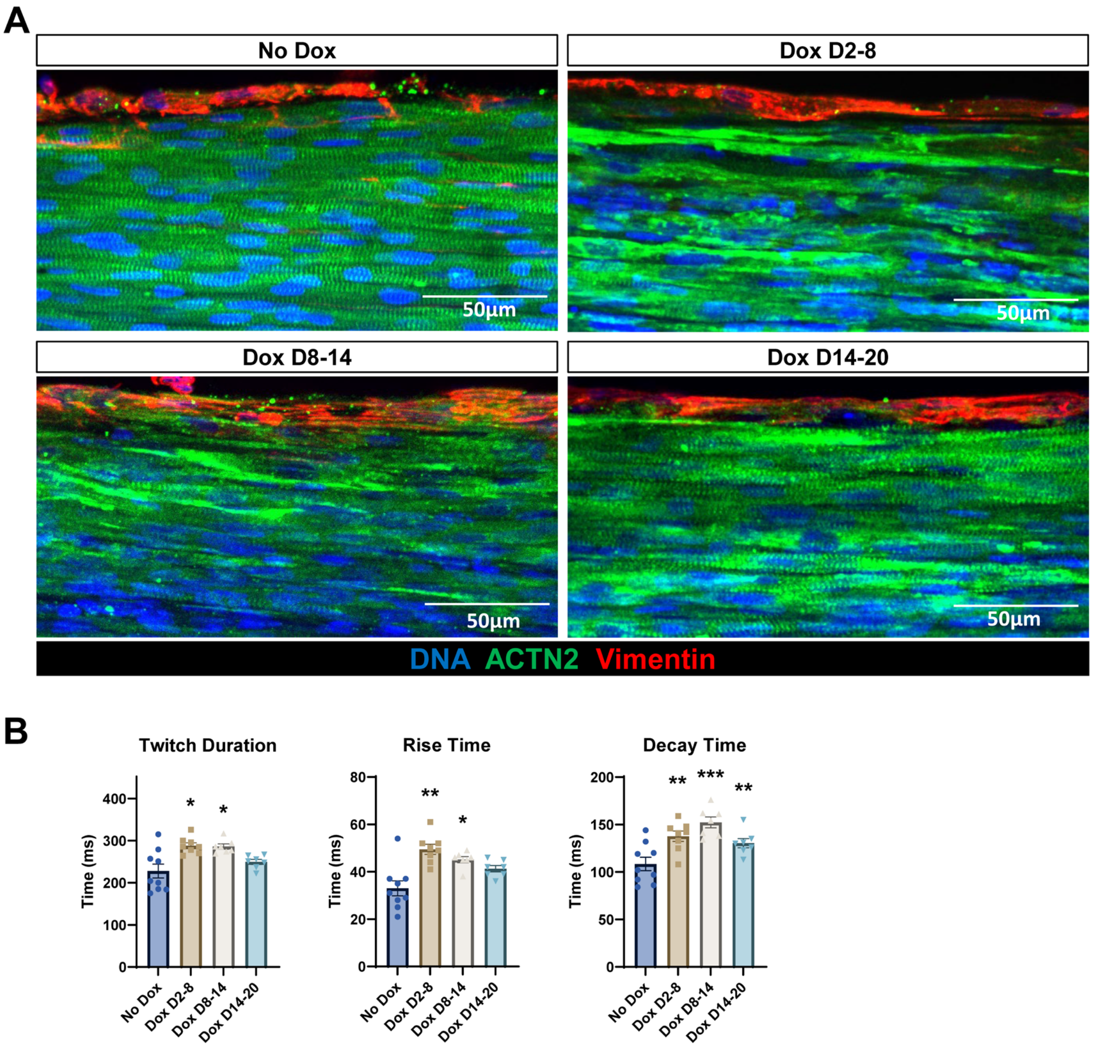

Figure S11. Effects of 6d Doxycycline Treatment on Sarcomere Structure and Twitch Kinetics of ECTs. (A-B) Representative whole-mount images showing sarcomere structure of ECTs (A) and quantification of twitch kinetics of tissues treated with doxycycline on the indicated days to induce caBRAF expression with a D20 endpoint (B). Data: n=7-9 ECTs (B). Column graphs showing individual data points, mean ± SEM. Line plots, mean + SEM. *p < 0.05, **p < 0.01, ***p < 0.001 vs. Ctrl LV+Veh. All experiments were repeated in N=3 independent ECT batches.

Figure S12

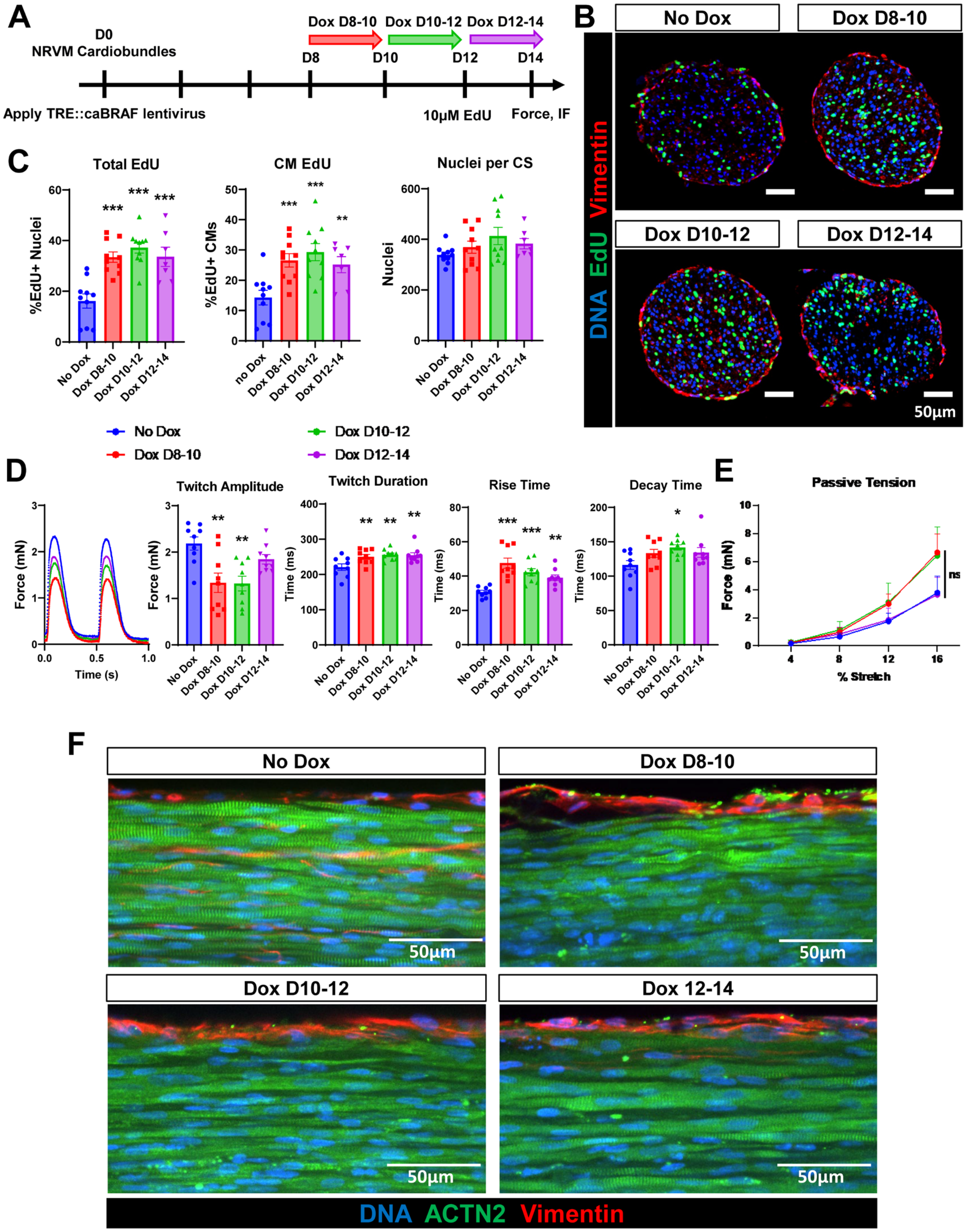

Figure S12. Brief Expression of caBRAF Reveals Rapid Increase in Cell Cycle Activity Followed by Functional Decline of ECTs. (A) Schematic of experimental design for (B-F). (B-F) Representative cross-section images of ECTs (B) and quantifications of cell cycle parameters (C), twitch amplitude and kinetics with representative force traces (D), and passive tension-length

relationships (E). (F) Representative whole-mount images showing sarcomere structure of ECTs. Data: n=9 ECTs (C,E), n=7-10 ECTs (D). Column graphs showing individual data points, mean ± SEM. Line plots, mean + SEM. $^{*}p < 0.05$, $^{**}p < 0.01$, $^{***}p < 0.001$ vs. indicated group. All experiments were repeated in N=3 independent ECT batches.

Figure S13

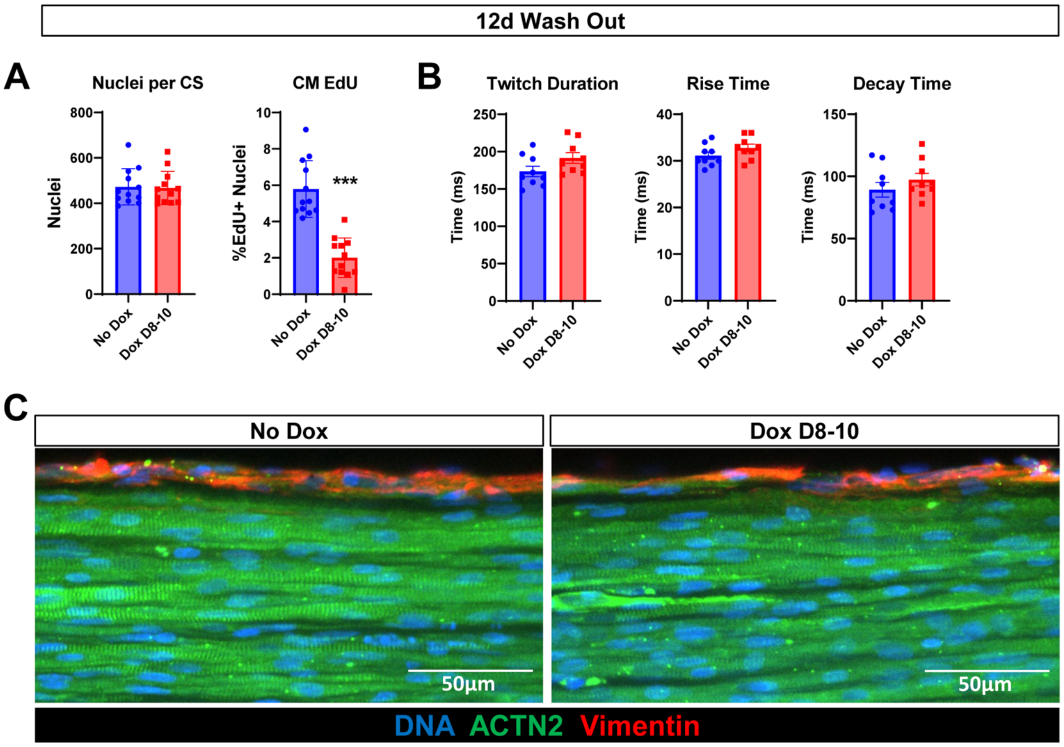

Figure S13. 2d Doxycycline Treatment Followed by 12d Washout Period Shows Reduced Cycling and no Effects on Twitch Kinetics of ECTs. (A-C) Additional analyses of ECTs treated with dox for 2d followed by a 12d washout showing quantified (A) cell cycling parameters and (B) twitch kinetics, as well as (C) representative whole-mount images of ECTs. Data: n=12 ECTs (A), n=9 ECTs (B). Column graphs showing individual data points, mean ± SEM. Line plots, mean + SEM. *p < 0.05, **p < 0.01, ***p < 0.001 vs. Ctrl LV+Veh. All experiments were repeated in N=3 independent ECT batches.

Figure S14

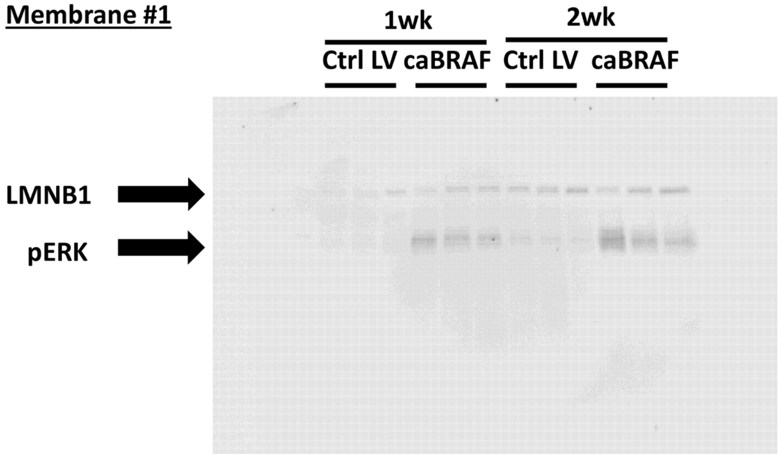
Membrane #1
1wk
2wk
Ctrl LV
caBRAF
Ctrl LV
caBRAF
LMNB1
pERK

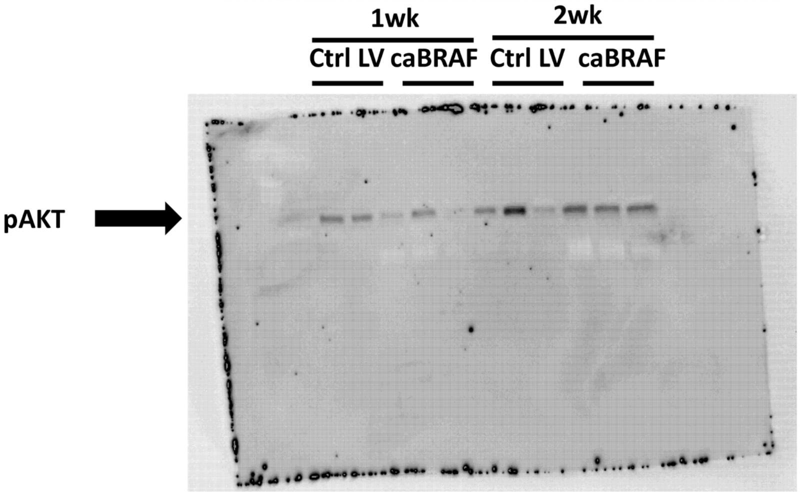
1wk
2wk
Ctrl LV
caBRAF
Ctrl LV
caBRAF
pAKT

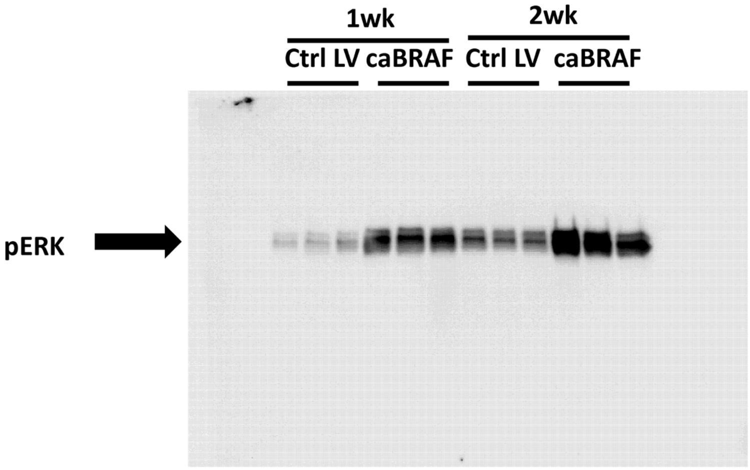
1wk
2wk
Ctrl LV
caBRAF
Ctrl LV
caBRAF
pERK

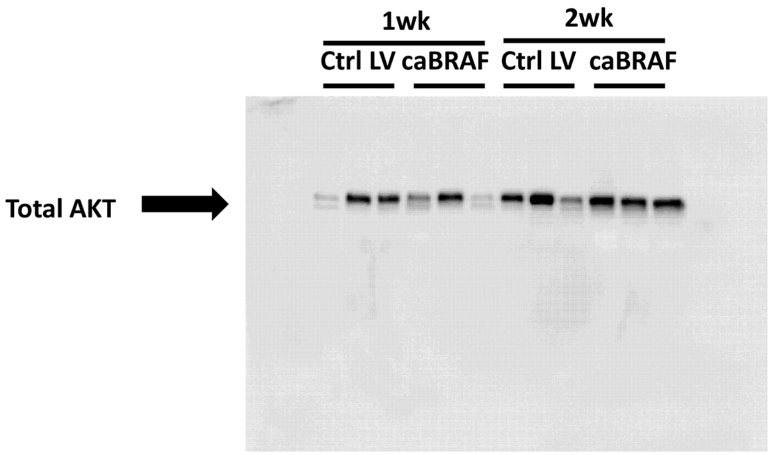
1wk
2wk
Ctrl LV
caBRAF
Ctrl LV
caBRAF
Total AKT

Membrane #2

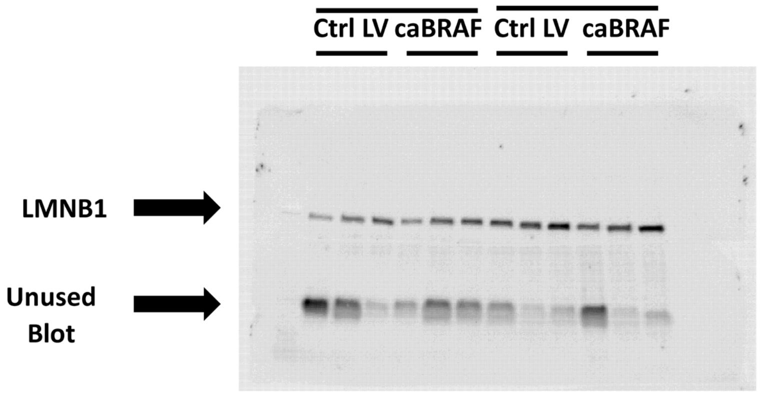
1wk
2wk
Ctrl LV
caBRAF
Ctrl LV
caBRAF
LMNB1
Unused Blot

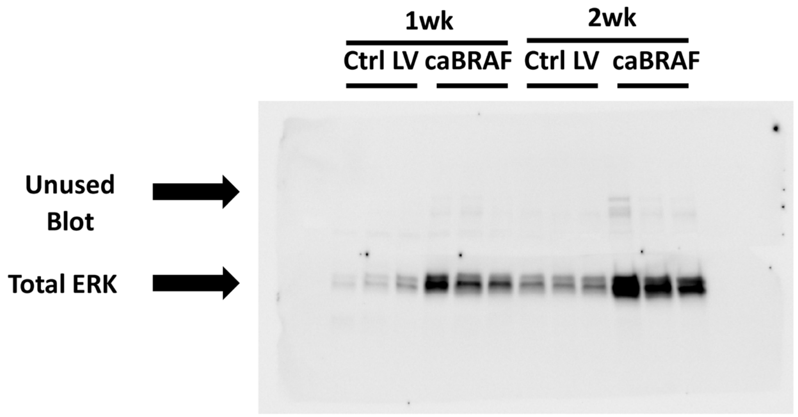
1wk
2wk
Ctrl LV
caBRAF
Ctrl LV
caBRAF
Unused Blot
Total ERK

Membrane #3

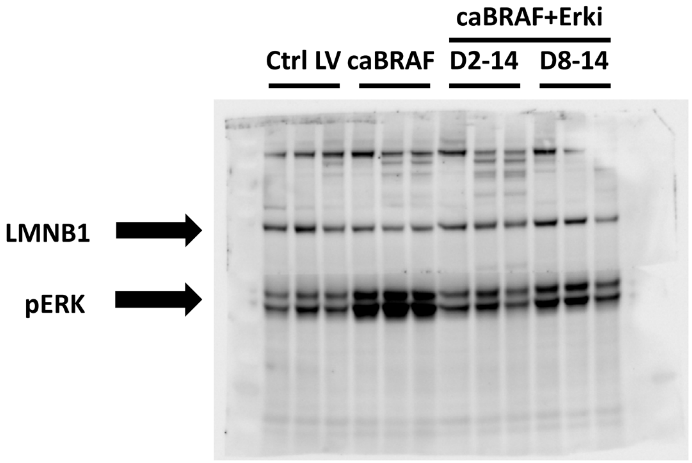
caBRAF+Erki
Ctrl LV
caBRAF
D2-14
D8-14
LMNB1
pERK

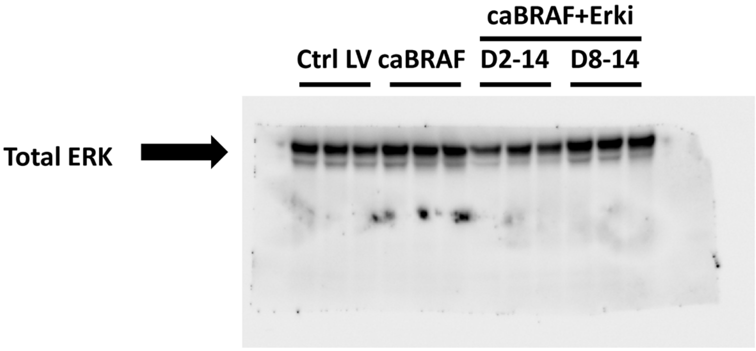
caBRAF+Erki
Ctrl LV
caBRAF
D2-14
D8-14
Total ERK

Membrane #4

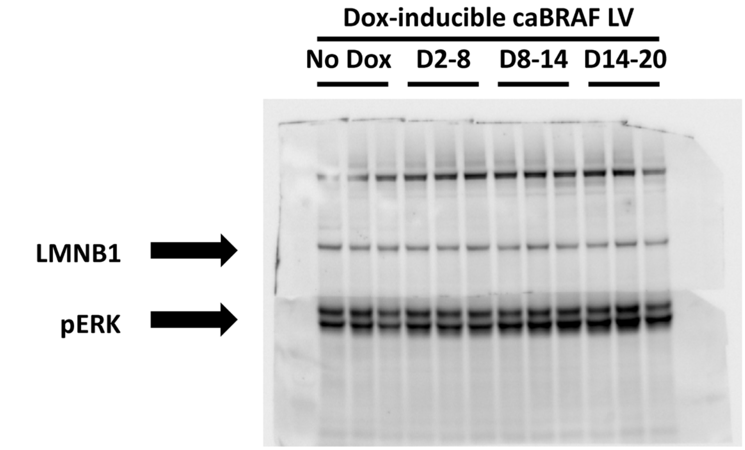

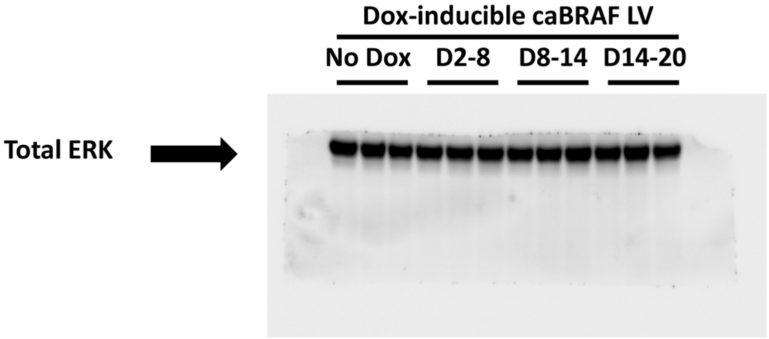

Membrane #5

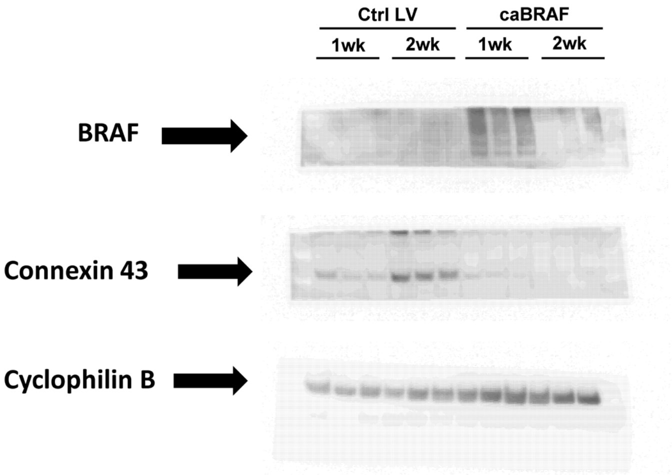

Figure S14. Uncropped, labeled Western Blots used in this manuscript.

Table S1

Reagent type	Reagent	Source	Catalog number	Additional Information
Antibody	Anti-Cardiac Troponin T (Rabbit polyclonal)	Abcam	ab45932	FC (1:200)
Antibody	Anti-Sarcomeric Alpha Actinin (Mouse monoclonal)	Sigma	A7811	IF (1:200)
Antibody	Cleaved Caspase-3 (Asp175) (Rabbit polyclonal)	Cell Signaling Technology	9661	FC (1:800)
Antibody	Mouse IgG1, kappa monoclonal [15-6E10A7] - Isotype Control	Abcam	ab170190	FC (1:2000)
Antibody	Phospho-p44/42 MAPK (Erk1/2) (Thr202/Tyr204) (Rabbit polyclonal)	Cell Signaling Technology	9101	WB (1:1000)
Antibody	p44/42 MAPK (Erk1/2) (Total Erk) (Rabbit polyclonal)	Cell Signaling Technology	9102	WB (1:1000)
Antibody	Rabbit IgG, polyclonal - Isotype Control	Abcam	ab37415	FC (1:2000)
Antibody	Recombinant Anti-AKT1 (phospho S473) [EP2109Y] (Rabbit monoclonal)	Abcam	ab81283	WB (1:5000)
Antibody	Recombinant Anti-AKT1 + AKT2 + AKT3 [EPR16798] (Rabbit monoclonal)	Abcam	ab179463	WB (1:10000)<br>IF (1:100)
Antibody	Recombinant Anti-Vimentin [EPR3776] (Rabbit monoclonal)	Abcam	ab92547	IF (1:500)
Antibody	Anti-alpha smooth muscle Actin antibody (Rabbit polyclonal)	Abcam	ab5694	IF (1:200)
Antibody	Anti-N Cadherin antibody (Rabbit polyclonal)	Abcam	ab18203	IF (1:300)
Antibody	Braf antibody (Rabbit Polyclonal)	ThermoFisher	PA5-81931	WB (1:1000)
Antibody	Connexin 43 antibody (Rabbit polyclonal)	Abcam	ab11370	WB (1:1000)
Antibody	Cyclophilin B antibody (Rabbit polyclonal)	Abcam	ab16045	WB (1:1000)
Chemical compound	SCH772984	Cayman Chemical	19166	
Chemical compound	Mitomycin C	Sigma	M4287	
Chemical compound	Phalloidin (AF488)	Thermo Fisher	A12379	
Chemical compound	Hydrazine Sulfate	Sigma	216046	
Chemical compound	B-Nicotinamide adenine dinucleotide hydrate (NAD+)	Sigma	N3014	
Chemical compound	Lactate Dehydrogenase	Sigma	L2500	
Chemical compound	Doxycycline Hyclate	Sigma	D9891	
Recombinant Protein	Recombinant Human OPG	Peprotech	450-14	
Recombinant Protein	Recombinant Human GDF-15/MIC-1	Peprotech	120-28C	
Commercial assay kit	Click-iT® EdU Alexa Fluor® 488 Imaging Kit	Thermo Fisher	C10337	
Commercial assay kit	Amplex™ Red Glucose/Glucose Oxidase Assay Kit	Thermo Fisher	A22189	
Commercial assay kit	Proteome Profiler Rat XL Cytokine Array	R&D Systems	ARY030	

Table S1. Antibodies and reagents used in this manuscript.

Data S1. Raw data points used to generate figures in this manuscript.
